# Mitochondria play an essential role in the trajectory of adolescent neurodevelopment and behavior in adulthood: evidence from a schizophrenia rat model

**DOI:** 10.1038/s41380-022-01865-4

**Published:** 2022-11-15

**Authors:** Hila M. Ene, Rachel Karry, Dorit Farfara, Dorit Ben-Shachar

**Affiliations:** 1 Lab of Psychobiology, R.& B. Rappaport Faculty of Medicine, Technion- Israel Institute of Technology, Haifa, Israel; 2 Department of Neuroscience, R.& B. Rappaport Faculty of Medicine, Technion – Israel Institute of Technology, Haifa, Israel; 3 Department of Immunology, R.& B. Rappaport Faculty of Medicine, Technion - Israel Institute of Technology, Haifa, Israel

**Keywords:** Neuroscience, Molecular biology

## Abstract

Ample evidence implicate mitochondria in early brain development. However, to the best of our knowledge, there is only circumstantial data for mitochondria involvement in late brain development occurring through adolescence, a critical period in the pathogenesis of various psychiatric disorders, specifically schizophrenia. In schizophrenia, neurodevelopmental abnormalities and mitochondrial dysfunction has been repeatedly reported. Here we show a causal link between mitochondrial transplantation in adolescence and brain functioning in adulthood. We show that transplantation of allogenic healthy mitochondria into the medial prefrontal cortex of adolescent rats was beneficial in a rat model of schizophrenia, while detrimental in healthy control rats. Specifically, disparate initial changes in mitochondrial function and inflammatory response were associated with opposite long-lasting changes in proteome, neurotransmitter turnover, neuronal sprouting and behavior in adulthood. A similar inverse shift in mitochondrial function was also observed in human lymphoblastoid cells deived from schizophrenia patients and healthy subjects due to the interference of the transplanted mitochondria with their intrinsic mitochondrial state. This study provides fundamental insights into the essential role of adolescent mitochondrial homeostasis in the development of normal functioning adult brain. In addition, it supports a therapeutic potential for mitochondria manipulation in adolescence in disorders with neurodevelopmental and bioenergetic deficits, such as schizophrenia, yet emphasizes the need to monitor individuals’ state including their mitochondrial function and immune response, prior to intervention.

## Introduction

Mitochondrial function and dynamics are particularly important during early brain neurodevelopment especially in processes of neurogenesis, neuronal differentiation, migration and maturation [1, 2]. In addition, ample evidence highlights the role of mitochondria in mature neurons, in neurite outgrowth, structural plasticity of dendritic spines and neurotransmitter release [3]. Neurons’ reliance on mitochondria is unique by the virtue of their high need for energy and dependence on Ca^2+^ homeostasis for their activity and survival, two core functions of mitochondria. In addition, mitochondria are major hubs of multiple cellular processes including redox balance maintenance, apoptosis, steroid production and heme biosynthesis. They also provide metabolites that serve as building blocks for macromolecules and modulate epigenetic processes [3]. It is therefore no wonder that mitochondrial impairments are repeatedly evidenced in a variety of neurodevelopmental disorders including Rett syndrome, autism spectrum disorder and schizophrenia (SZ) [4, 5]. In SZ, we and others have demonstrated multiple mitochondria impairments, including structural, genetic, molecular and biochemical deficits, converging at abnormal cellular O_2_ consumption and ATP production [4, 6, 7].

SZ is conceptualized as a neurodevelopmental disorder, in which symptoms are manifested in early adulthood following subtle prenatal disturbances in brain development that substantially accelerate during adolescence, a period of progressive maturational processes [8]. Abnormalities in neuronal morphology, connectivity and transmission of multiple neural systems have been observed in SZ [9]. 2D and 3D cultures of developing induced pluripotent stem cells (iPSCs) have captured deficits in neurogenesis and neuronal differentiation in the disorder [10, 11]. In-vivo experimental models, such as the maternal immune activation (MIA) models, have significantly added to our understanding of SZ neurodevelopmental-related deficits [2, 12]. Preclinical MIA models have unraveled the effects of the maternal immune response on offspring brain development including impaired neurogenesis, delayed myelination and axonal development, decreased hippocampal and striatal volumes and increased lateral ventricular volume [13]. MIA studies have also demonstrated functional and behavioral changes associated with SZ such as abnormalities in latent inhibition, prepulse inhibition, novel object recognition and increased responses to amphetamine [14]. In addition, mitochondrial dysfunction has been observed in MIA models, including deficits in mitochondria membrane potential (Δψm), enzyme activity, transcript levels of the respiratory complexes’ subunits and mitochondrial morphology [2, 15, 16] turning MIA into an attractive model for studying the link between mitochondria and neurodevelopment. One plausible way to modulate mitochondrial function is by transplantation of healthy mitochondria. During the last decade, it was reported that mitochondria transplantation induces beneficial effects in various pathophysiological conditions associated with mitochondrial dysfunction [15, 17–20]. In the context of the CNS, few studies, including ours, have shown that mitochondria transplantation ameliorated deficits in cognitive and locomotor behaviors in rodent models of neuro-psychiatric disorders such as SZ, Alzheimer’s and Parkinson’s diseases and cerebral ischemia-reperfusion injury [15, 17, 20, 21]. In SZ-derived iPSCs, transplanted mitochondria promoted neuronal differentiation and survival [15].

Taken together, the hitherto data suggest an association between mitochondria and CNS function. Yet, to the best of our knowledge, none of the previous studies shows a mechanism-based causative link between mitochondria and normal adolescent neurodevelopment, in which refining processes of brain structure and circuitry are taking place [22]. Here we provide empirical evidence for a host-dependent reciprocal link between mitochondria and CNS functions in the MIA-SZ model and in healthy control rats, by transplanting healthy mitochondria into the medial prefrontal cortex (mPFC), which parallels the dorsolateral prefrontal cortex, a major dysfunctional site in SZ patients [23, 24]. We show that interfering with host mitochondrial homeostasis, at a critical time-point of cortical development, prevented SZ pathogenesis, yet shifted the normal equilibrium towards abnormality. We demonstrate almost opposite trajectories of change, initiating from altered mitochondrial function and inflammatory response, leading to long-lasting changes in proteome, neurotransmitter turnover, neuronal morphology and ultimately behavior in adult offspring. An inverse shift in mitochondrial function due to interference with their original state was also observed in SZ and healthy subjects-derived lymphoblastoid cell lines (hLCLs) following mitochondria transplantation.

## Materials and methods

Detailed description of the study’s Methods is provided in Supplementary Method Section. All samples were coded and analyzed with the experimenter blinded as much as possible to the origin of the samples.

### MIA and hLCLs SZ-models

The viral mimic polyinosinic:polycytidylic acid (Poly I:C) MIA model was obtained as described previously [12, 15] (For details see Supplementary Methods). All experimental protocols conformed to the guidelines of the Technion Institution and NIH Institutional Animal Care and Use Committees.

hLCLs were derived from DSM-IV diagnosed SZ patients (11 males, 7 females, average age 40.1 ± 2.5 years old, range 22–61 years) and healthy control (HC) subjects with no prior history of psychiatric illness (12 males, 8 females, average age 42.2 ± 2.2 years old, range 22–60 years). hLCLs were kindly obtained from Prof. Richard P Ebstein, Beer-Sheva Mental Health Center, Israel and from Prof. Peter Zill, Psychiatry Center, Ludwig-Maximilians-University of Munich, Germany. The study was approved by the both Ceneters’ Helsinki Committees. All participants gave a written informed consent. hLCLs were cultured as previously described [25].

### Mitochondria isolation and transplantation

Active mitochondria were isolated from naïve rat brains [26] and from SZ and HC-derived hLCLs [25], on a Percoll gradient, the final pellet was suspended in ice-cold 10 mM Tris–HCl buffer (pH 7.4) containing 0.25 M sucrose and 1 mM dehydroascorbic acid (DHA) (vehicle), and their purity, integrity and activity were analyzed as described previously [26].

Intracerebral injection of mitochondria or vehicle was performed in four independent experiments (*n* = 150 animals, for details see legends to the figures and Supplementary Methods) on PND 34, as described previously [15]. Four experimental groups were obtained: (1) saline and (2) Poly I:C prenatally exposed offspring injected with vehicle (SV and PV, respectively), (3) saline and (4) Poly I:C prenatally exposed offspring transplanted with mitochondria (SM and PM, respectively).

hLCLs were incubated at 37 °C overnight, with vehicle or active mitochondria (50 µg protein/10^6^ cells) isolated from SZ- or HC-hLCLs. The experimental array composed six groups: (1) HC-cells+HC-mitochondria (2) HC-cells+SZ-mitochondria (3) HC-cells+vehicle (4) SZ-cells+HC-mitochondria (5) SZ-cells+SZ-mitochondria (6) SZ-cells+vehicle.

For tracing mitochondria cell entrance, isolated active mitochondria were incubated with 50 nM MitoTracker-Orange CMTMRos dye (Invitrogen, ThermoFisher Scientific) on ice for 20 min followed by two washing steps. Stained mitochondria were intracerebrally injected to Poly I:C and saline offspring and their incorporation into cells was followed 3 h after transplantation by Zeiss LSM880 confocal microscope with X63 Plan Apochromat oil objective. Estimation of mitochondria entrance into cells was assessed by the intensity of cellular MitoTracker-Orange/cell volume using an in-house Python script.

### Behavior testing

All tests were conducted during the dark cycle at the following order: social recognition, spontaneous locomotor activity in a novel environment, and amphetamine-induced activity. Behaviors were evaluated in the same animals (6–8 animals/group) in adulthood (PND > 90) when symptoms are fully manifested, using the Ethovision 7 software (Noldus, Wageningen, The Netheralnds).

*Social recognition in a novel environment:* was evaluated on PND 91–93 as described previously [27]. The test rat was free to explore all three compartments and time spent in each was recorded for 10 min. Social recognition index was calculated as follows: (F-NF)/(F + NF); F = time spent in the chamber with a familiar rat; NF = time spent in the non-familiar empty chamber.

*Spontaneous locomotor activity in a novel environment:* On PND 100, rats were placed in novel dark boxes (50 cm wide X 70 cm long X 40 cm high) for 30 min during which the distance traveled (cm) by each rat was recorded in 6 min blocks and the distance each rat traveled during 30 min was analyzed.

*Amphetamine-induced activity:* Immediately after spontaneous locomotor activity test, each of the previous rats was injected with amphetamine (1.0 mg/kg; Sigma-Aldrich, Israel) and replaced into the same experimental boxes and the distance traveled (cm) was recorded in 6 min blocks during 30 min.

### Histological and biochemical measurements

Animals were euthanized at three time-points: two (PND 36) and seven (PND 41) days after transplantation, and in adulthood on PND 120, one week after the end of behavioral testing. For immunofluorescence and histochemistry, animals were anesthetized by Ketamine/Xylazine cocktail and intracardially perfused with 4% paraformaldehyde (PFA) or PBS. Brains were immediately dissected, snapped-frozen with liquid nitrogen and stored at −80 °C until use. Coronal brain sections (13 µm thick) including the mPFC and primary motor area (M1: AP 1.7 to 2.7 from bregma) were cut at −25 °C using a cryostat (Leica, Germany). For qRT-PCR, high-pressure liquid chromatography (HPLC) and proteomics, animals were decapitated under anesthesia. The mPFC (AP 2.0 to 3.4; ML 1.1; DV 5 from bregma) was dissected and stored at −80 °C until use.

### HPLC

Monoamines’ concentrations were measured on PND 120 in frozen mPFC samples (*n* = 5–8 animals/group). Norepinephrine (NE), dopamine (DA), its metabolites 3,4-dihydroxyphenylacetic acid (DOPAC) and homovanillic acid (HVA), serotonin (5-HT) and its metabolite 5-hydroxyindoleacetic acid (5-HIAA) and DA and 5-HT turnover rates were assessed by HPLC with an electrochemical detector and analyzed as described previously [28].

### Golgi-Cox staining

On PND 120, animals (*n* = 4–5 animals/group) were deeply anesthetized with isoflurane, decapitated and their brains immediately dissected and processed using the FD Rapid GolgiStain kit (FD Neurotechnologies, USA) according to the manufacturer’s instructions. Image acquisition, dentrite reconstraction and spine density assessments were done as previously reported [29].

### Immunofluorescence

Fixated (4% PFA) and permeabilized (0.5% Triton X-100) mPFC slices were blocked and incubated with primary and secondary antibodies (Supplementary Methods) and then mounted with Dapi Flouromount-G (SouthernBiotech). Slides were scanned using the Zeiss LSM880 confocal microscopy with a x63 Plan-Apochromat oil objective for NeuN and GFAP, or by 3D Histech Pannoramic 250 Flash III slide scanner with a Plan-Apochromat x20 objective connected to an pco.edge 4.2 camera for c-Fos and Iba1 assessments. Nuclei c-Fos^+^ and cellular Iba1^+^ were analyzed using in-house Python scripts. c-Fos activation was calculated by nuclei c-Fos total intensity/the total number of Dapi stained nuclei. Microglia density in the mPFC and the adjacent M1 was calculated by the number of Iba1^+^ cells/tissue area.

### Mitochondria functional parameters

*Succinate dehydrogenase (SDH) and cytochrome c oxidase (COX) ex-vivo activities* were assesses on PND 36 and PND 41 in mPFC frozen sections as described previously [30]. Brain sections were scanned using the above mentioned slide scanner connected to an Adimec Q12A180 camera. Enzyme activity was analyzed using an in-house Python script and calculated by dividing blue SDH or brown COX stain integrated intensity/tissue area.

*ROS production* was measured on PND 36 and PND 41 in mPFC frozen sections using the redox-sensitive dye 6-carboxy-2’,7’-dichlorodihydrofluorescein diacetate (CM-H_2_DCFDA) (Invitrogen, USA) as described previously [31]. For autofluorescence, sections were exposed to PBS. Twenty five images were immediately taken by Zeiss Axio Imager Z2 upright microscope (Zeiss, Germany) with x40 EC Plan-Neofluar objective. H_2_DCFDA puncta were quantified using an in-house Python script. ROS levels were calculated by the number of oxidized H_2_DCFDA puncta/tissue area.

*Mitochondrial basal respiration and its inhibition by DA in hLCLs:* Complex I (CoI) driven oxygen consumption rate and its inhibition by DA was measured as described previously [32].

### qRT-PCR analysis

RNA was extracted from frozen brain samples of the four experimental groups and naïve Poly I:C and saline offspring and qRT-PCR was performed as described previously [33]. Experimental samples were quantified using the ΔΔCt method against *Gapdh*, which was not affected by treatment, and against the averaged ΔCt of four saline naïve offspring. Naïve samples were quantified using the ΔCt method against *Gapdh*. Primers’ data are listed in Supplementary Table 1.

### Proteomics and phosphoproteomics

Protein extraction and trypsinization as well as phosphopeptides enrichments of mPFC samples were performed and the resulted peptides were analyzed by LC-MS/MS using a Q-Exactive Plus mass spectrometer (Thermo) fitted with a capillary HPLC (easy nLC1000, Thermo-Fisher). For data analysis and bioinformatics see Supplementary Methods.

### Statistics

Results were analyzed for normal distribution by Kolmogorov-Smirnov test. Two groups were compared by a Student’s *t*-test or paired *t*-test, multiple comparisons by one-way ANOVA followed by Tukey’s *posthoc* test or two-way ANOVA followed by simple effect analysis. Linear discriminant analysis (LDA) was done for the cytokine expression. Multivariate correlation matrix was applied and pairwise linear correlation coefficient was assessed by Pearson’s correlation, *r* ≥ |0.7 | , *P* ≤ 0.05 was considered significant. Results are expressed as mean ± s.e.m. *P* ≤ 0.05 was considered statistically significant. GraphPad Prism 9.1.1 and IBM SPSS statistics 24 software were used.

## Results

### Behavior, neuronal morphology and monoamines alterations in adulthood

**Fig. 1:**
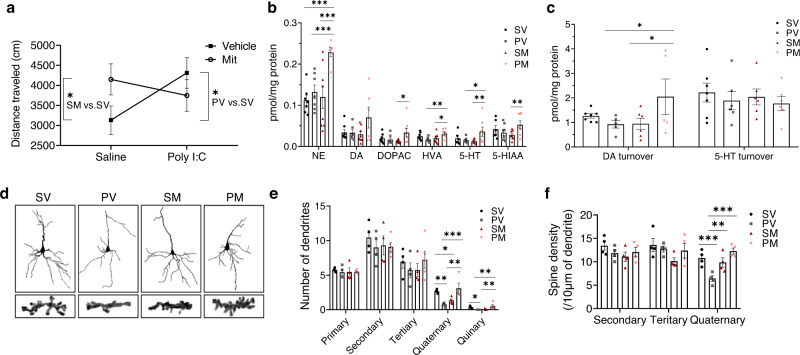
Mitochondria transplantation into the mPFC in adolescence affects mPFC-dependent stress-induced spontaneous locomotor activity, neurotransmitter levels and neuronal outgrowth, assessed by dendritic branching and spine density of II/III layer pyramidal neurons, in adulthood (PND > 90). **a** Significant differences in distance traveled of spontaneous locomotor activity in a novel environment between the experimental groups (“model X transplantation” interaction (*F*(1,25) = 4.274, *P* < 0.049). Both PV and SM showed increased locomotor activity as compared to SV rats (*P* > 0.035 and *P* < 0.05, respectively). PM locomotor activity was attenuated towards SV activity levels (*P* = 0.276), yet did not reach a significant difference from PV (*P* = 0.331). *N* = 6–8 rats/group. **b** Two-way ANOVA identified significant “model X transplantation” interaction effects for the monoamines’ neurotransmitters NE (*F*(1,24) = 5.163, *P* < 0.032), DOPAC (*F*(1,26) = 4.751, *P* < 0.039), HVA (*F*(1,24) = 6.811, *P* < 0.015), 5-HIAA (*F*(1,25) = 4.528, *P* < 0.038), and a marginal interaction for 5-HT levels (*F*(1,21) = 3.714, *P* < 0.06). In PM rats, simple effect analysis showed significant increases in NE (at least *P* < 0.003 compared to all other groups), DOPAC (PM vs. SM, *P* < 0.038), HVA (PM vs. PV, *P* < 0.014; PM vs. SM, *P* < 0.034), 5-HT (PM vs. PV, *P* < 0.05; PM vs. SM, *P* < 0.029) and in 5-HIAA levels (PM vs. SM, *P* < 0.026). *N* = 5–8 rats/group. **c** Significant interaction for DA turnover (*F*(1,19) = 4.356, *P* < 0.05) due to a significant increase in PM as compared to PV (*P* < 0.043) and to SM (*P* < 0.039). No significant differences were observed for 5-HT turnover. *N* = 5–8 rats/group. **d** Representative images of computer-generated reconstruction of Golgi-cox-impregnated pyramidal neurons and spines’ images along 10 µm segment of quaternary dendrites of all groups. **e** Number of primary, secondary, tertiary, quaternary and quinary order dendrites. One-way ANOVA revealed significant differences in quaternary (*F*(3,12) = 6.70, *P* < 0.007) and quinary (*F*(3,12) = 3.66, *P* < 0.044) dendrites between groups. PV showed a decreased number of quaternary (*P* < 0.008) and no quinary (*P* < 0.048) dendrites as compared to SV, while transplanted mitochondria increased both (*P* < 0.002 and *P* < 0.015, respectively) in PM as compared to PV group with no change from SV. In SM rats, mitochondria transplantation decreased the number of quaternary (*P* < 0.043) branching as compared to SV. *N* = 4–5 rats/group. **f** Number of secondary, tertiary and quaternary spine density per 10 µm dendritic segment. One-way ANOVA showed significant differences (*F*(3,12) = 10.1, *P* < 0.001) in spine density of quaternary dendrites between groups. Lower quaternary spine density was observed in PV as compared to SV rats (*P* < 0.001). Mitochondria transplantation increased spine number in PM as compared PV rats to reach SV number (PM vs. PV, *P* < 0.0002; PM vs. SV, *P* > 0.05), but had no effect in the SM group. *N* = 4 rats/group. All values are means ± s.e.m. *P < 0.05; **P < 0.03; ***P < 0.005.

Previously we have shown that transplantation of mitochondria into the mPFC in adolescence restores SZ-related selective attention deficit in PM, while provoking attention deficit in SM adult rats [15]. Here we further investigated the spreading of mPFC mitochondrial transplantation effects to various behavior-controlling brain networks. Hence we assessed SZ-related behaviors for which the mPFC, orbitofrontal cortex and the striatum are critical controlling areas [12, 34, 35]. Spontaneous locomotor activity in response to a novel environment, which has been linked with mPFC activity [36], was significantly (*P* < 0.035) increased in PV as compared to SV rats (Fig. 1a). Mitochondria transplantation reduced this hyperactivity in PM rats to similar levels of SV (PM vs. SV, *P* < 0.28). In contrast, in SM rats, it led to hyper-locomotion (SM vs. SV, *P* < 0.05) mirroring PV behavior. Social recognition and amphetamine-induced hyperactivity, primarily linked with orbitofrontal and striatal activities, respectively [37, 38], were not affected by mitochondria transplantation, and were impaired in both Poly I:C groups (Poly I:C vs. saline, *P* < 0.045 and *P* < 0.015, respectively) (Supplementary Fig. 1a, b). In line with the behavioral data, a significant (at least *P* < 0.05) increase in mPFC monoamines NE, HVA, 5-HT levels and in DA turnover was observed in adult PM as compared to the PV group, with NE elevated in comparison with all other groups (at least *P* < 0.003) (Fig. 1b, c). In SM rats, mitochondria transplantation had no effect on monoamines as compared to SV, yet NE, DOPAC, HVA, 5-HT, 5-HIAA and DA turnover were significantly (at least *P* < 0.039) decreased as compared to PM rats. No difference was observed between PV and SV rats, supporting previous findings in this model [39]. In the striatum, mitochondria transplantation had no effect on monoamines, including DA, which levels were increased in both PV and PM compared to both saline groups (*P* < 0.017) (Supplementary Fig. 1c, d). Striatal NE level was below detection threshold.

The behavioral together with the monoamines’ data suggest a localized effect of mitochondria transplantation, and were associated with structural changes in mPFC neurons 90 days post-transplantation (PND 125). In line with the latter, lower distance traveled in a novel environment was significantly correlated with higher quaternary spine density (*r* = −0.99, *P* < 0.01), and with lower levels of 5-HT (*r* = 0.96, P < 0.03) and NE (*r* = 0.72, *P* < 0.05) in SV rats. In addition, neuronal morphology showed correlations with mPFC monoamines. Namely, quaternary and quinary dendritic branching were negatively correlated with DA turnover (*r* = −0.997. *P* < 0.03) and positively correlated with DA levels (*r* = 0.908, *P* < 0.05), respectively. Between groups, significant differences were observed in quaternary (*P* < 0.007) and quinary (*P* < 0.044) dendritic branching of layer II/III pyramidal neurons with no significant differences in primary, secondary and tertiary dendrites number (Fig. 1d, e). Specifically, PV exhibited less quaternary dendritic branching (*P* < 0.008) and quinary branching was almost absent (*P* < 0.048) as compared to SV rats. Mitochondria transplantation significantly increased the number of both quaternary (*P* < 0.002) and quinary (*P* < 0.015) branching in PM as compared to PV rats, yet in SM decreased the number of quaternary (*P* < 0.043) dendrites as compared to SV rats. Enriched neuronal branching in PM was associated with a significant increase in quaternary dendrites spine density as compared to PV (*P* < 0.0002), which exhibited lower quaternary spine density as compared to all other groups (at least *P* < 0.008 for all comparisons) (Fig. 1d, f). SM quaternary spine density was not different from that of SV and PM. No difference was observed in branching and spine density of secondary and tertiary dendrites.

### Alterations in proteome and phosphoproteome in adulthood

**Fig. 2:**
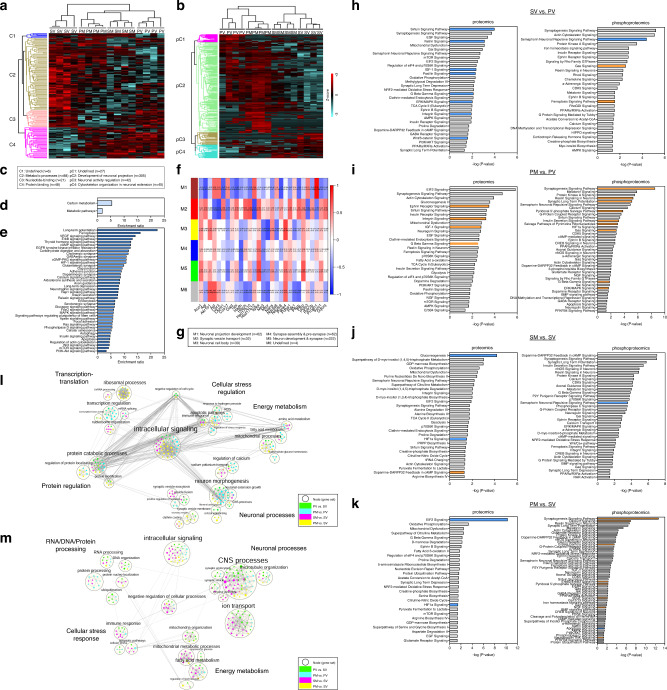
The effects of mitochondria transplantation on mPFC proteome and phosphorylation events in the four experimental groups (SV, PV, SM and PM) assessed in the adult offspring (PND > 120). **a**, **b** Unsupervised hierarchical clustering of proteins (**a**) and phosphorylation events (**b**) resulted in **a**, 153 ANOVA-significantly altered proteins segregated into four clusters (C1–4), three enriched for metabolic processes (C2; brown), nucleotide-binding (C3; pink) and protein binding (C4; magenta); and in **b** 423 ANOVA-significantly altered phosphorylation events segregated into four clusters (pC1-4), three enriched for development of neuronal projection (pC2; green), neuronal activity regulation (pC3; brown) and cytoskeleton organization in neuronal extension (pC4; turquoise). Distance estimation between samples’ proteins, assessed by Euclidean distances, showed that SV clustered separately from all other three groups, still PM profile distance was closer to SV than that of SM and PV. Scale bar: Z-scores −3 to +3. **c** Legend of dendrogram clusters of proteome (C1–4) and phosphoproteome (pC1–4) of the hierarchical clustering. Dendrogram cluster enrichment was performed using STRING analysis and defined by GO terms. **d**, **e** Over-representation (ORA) analysis of ANOVA-significant proteins (**d**) and phosphoproteins (**e**) yielded significant functional enrichments for carbon metabolic metabolism and neuronal signaling pathways, respectively. **f** Weighted gene correlation network analysis (WGCNA) of significant proteins enriched for metabolic pathways (traits) and ANOVA-significant phosphoproteins (modules; M1-M6) showed significant bi-weight mid-correlations (*r* > |0.5|, *P* ≤ 0.05) between 90% of the proteins and 1–3 phospho-modules. Each row corresponds to a module, each column to a trait; each cell contains the corresponding correlation and its *P* value. The table is color-coded by correlation according to the color legend (Pearson’s r). **g** Legend of the phosphoproteins modules (M1-M6), and phosphoprotein number in brackets, mainly enriched for neuronal and synaptic morphology and development by STRING analysis. **h**, **i**, **j**, **k** Canonical pathway enrichment analysis of significant proteins and phosphorylation events differentially regulated between SV and PV (**h**), PM and PV (**i**), SM and SV (**j**), and PM and SM (**k**) groups in adulthood (PND 120), using Ingenuity Pathway Analysis (IPA). Prediction of activation was determined by Z-scores: activation (orange) - Z-score > 2; inhibition (blue) - Z-score < −2; undefined direction (gray). Enrichment significance: –log (*P* value) > 1.3 (red line = threshold of significance). **l**, **m** Enrichment maps of the proteome (**l**) and phosphoproteome (**m**) depicting the distribution of core functional cellular processes differentially enriched in the four group comparisons (PV vs. SV, PM vs. PV, SM vs. SV and PM vs. SV), using Gene-Set Enrichment Analysis (GSEA) and Cytoscape visualization. Each node (small circle) represents a distinct pathway; edges (gray lines) represent the number of genes overlapping between two pathways, determined by the coefficient of similarity. (**a**–**m**) *N* = 4 rats/group.

Next, we studied proteome and phosphoproteome, possibly underlying the behavioral and neuronal changes induced by transplanted mitochondria. A total of 3647 proteins and 4803 phosphorylated proteins (FDR < 0.01) were identified. Unsupervised hierarchical clustering of the significantly different proteins (*n* = 153) and protein phospho-sites (*n* = 419) distinguished between the four experimental groups and indicated the highest dissimilarity between SV and PV groups (Fig. 2a, b). Notably, a high similarity of proteome profile was observed between SV and PM, while that of SM was more similar to PV than to SV. Specifically, protein expression in the major metabolic processes cluster (C2) was increased in SV and PM, while decreased in PV and SM (Fig. 2a, c). In the phosphoproteome profile, the most profound differences between groups were observed in neuronal projection development (pC2) and cytoskeleton organization in neuronal extension (pC4) clusters (Fig. 2b, c), with PM showing the highest, while PV the lowest phosphorylation events in pC2 and pC4 clusters, respectively.

Pathway enrichment analysis of the significantly different proteins and phosphosites identified carbon metabolic pathways in the proteome (*n* = 30 proteins), while neuron synapse and post-synaptic signaling pathways (*n* = 52 phosphoproteins) in the phosphoproteome (Fig. 2d, e). Significant bi-weight mid-correlations (*r* > |0.5 | , *P* ≤ 0.05) were observed between 90% of the significantly different metabolic proteins and 1–3 phospho-modules out of five eigenprotein modules obtained by Weighted Correlation Network Analysis (WGCNA) (Fig. 2f, g), suggesting that alterations in neuronal function and development are closely linked with carbon metabolic pathways.

Deregulations mainly in energy metabolism and neuronal function-related pathways were also identified upon analyzing the following comparisons: PV vs. SV; PM vs. PV; PM vs. SV; SM vs. SV groups by Ingenuity Pathway Analysis (IPA) (Fig. 2h–k and Supplementary Tables 2 and 3). Pathways reaching inhibition/activation prediction (−2 > z-score>2) in the proteome and phosphoproteome analyses were mostly inhibited in PV vs. SV (sirtuin, netrin, integrin, IGF-1, Gβγ, Erk/MAPK, Wnt/β-catenin and semaphorin neuronal-repulsive pathways) (Fig. 2h), while activated in PM vs. PV (ephrin receptor, integrin, IGF-1 and Gβγ, synaptogenesis, reelin, LTP, Erk/MAPK, pyridoxal 5’-phosphate salvage, insulin secretion and pyrimidine ribonucleotides salvage pathways) (Fig. 2i). An interesting exception in PV vs. SV comparison was the activation of ferroptosis signaling, associated with deleterious ROS accumulation [40].

SM vs. SV comparison showed inhibition of gluconeogenesis, which correlates with enhanced glycolysis and mitochondrial deficits [41], and of semaphorin neuronal-repulsive signaling pathways, while activation of dopamine-DARPP32 feedback in cAMP signaling (Fig. 2j), possibly leading to an imbalance in dopaminergic, glutamatergic and GABAergic cellular signaling [42]. In PM vs. SV comparison, transplantation led to inhibition of apoptosis signaling, yet activation of various pathways vital for neural circuities, neurotransmission and synaptic plasticity (synaptogenesis, Ca2+, Gβγ, ErbB2-ErbB3, PLP salvage, VEGF, NGF, and Neurotrophin/TRK pathways) [43–45] (Fig. 2k). These hyper-shifted changes in PM may be required in order to reach recovery from the abnormal pattern. Puzzlingly, eIF2 signaling, essential for protein synthesis and affected by various stressors, was inhibited. In both PM and SM vs. SV, HIF-1α signaling, a major pathway in the hypoxia-ischemia response, which has been implicated in SZ [46–48], was inhibited (Fig. 2j, k), suggesting a long-lasting effect of mitochondrial transplantation on the modulation of oxygen saturation.

To identify global proteomics trends between the groups, proteins and phosphoproteins were analyzed for pathway enrichment (Fig. 2l, m and Supplementary Tables 4 and 5). Proteome data clustered into six core functions; neuronal processes, energy metabolism, cellular stress regulation, intracellular signaling, transcription-translation and protein regulation (Fig. 2l). Phosphoproteome clustered into similar five core functions; neuronal processes, energy metabolism, cellular stress response, intracellular signaling and RNA/DNA/protein processing (Fig. 2m). The differentially enriched pathways in the proteome were predominantly observed in PM vs. PV and PM vs. SV comparisons across all core functions, whereas in phosphoproteome in SM vs. SV and PV vs. SV. More specifically, according to proteome Normalized Enrichment Scores (NES) scores (Supplementary Table 4), mitochondria transplantation to Poly I:C rats caused an upregulation of pathways in all six core functions compared to PV. However, compared to SV, a more complex effect was observed, as intracellular signaling, neuronal processes and energy metabolism core functions were upregulated, while protein regulation, transcription-translation and cellular stress regulation were downregulated in PM, suggesting a new equilibrium state in PM mPFC enabling the restoration of behavioral response and neuronal outgrowth. In the phosphoproteome analysis, enrichments indicated an alteration in phosphorylation status leading to either activation or inhibition of the pathways composing the core functions. Upregulated phosphorylation in SM was observed in all core functions except for energy metabolism as compared to SV, whereas in PV alteration across core functions was inconsistent as compared to SV, with an increase, no change or decrease (Supplementary Table 5). In all, these data further suggest a disparate global effect of mitochondrial transplantation depending on disease/health state.

### Acute effects of mitochondria transplantation in adolescence

**Fig. 3:**
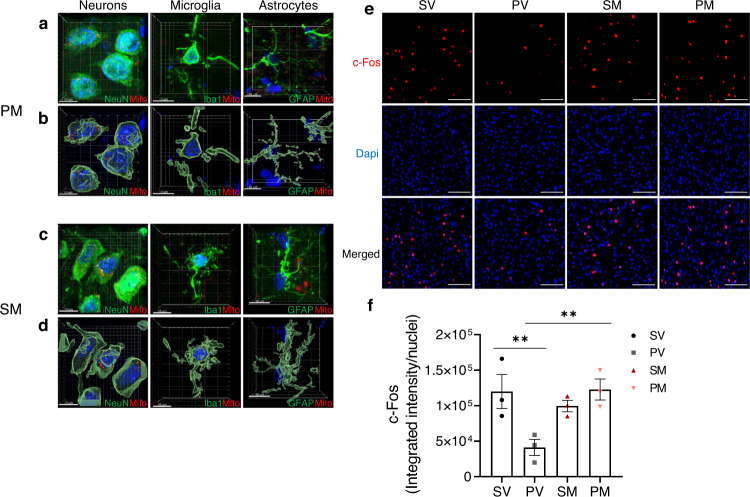
Mitochondria cell entrance and neuronal activation. **a**–**d** Representative confocal images of MitoTracker-stained mitochondria (red) 3 h after transplantation in the mPFC of PM (**a**, **b**) and SM (**c**, **d**) rats. **a**, **c** 3D presentations of integrated confocal scans and **b**, **d** Imaris modelizations of cells depicted in (**a**) and (**c**). Neurons were stained with NeuN (green), microglia with Iba1 (green) and astrocytes with GFAP (green). Scale bar: 10 µm. **e** Representative images of immunofluorescence staining of c-Fos, the early nuclear marker for neuronal activation, in the mPFC of the four experimental groups at two days after transplantation. Scale bar: 100 µm. **f** Quantification of c-Fos integrated intensity at two days after transplantation. One-way ANOVA showed a significant difference between groups (*F*(3,8) = 5.81, *P* = 0.020). In PM rats, mitochondria enhanced the reduced c-Fos activity of PV to normal levels (PV vs. SV, *P* < 0.031; PM vs. PV, *P* < 0.026). c-Fos activation was measured as c-Fos fluorescence intensity (red) co-localized with Dapi (blue) divided by the number of total nuclei. *N* = 3 rats/group; 3–6 sections/rat; *N* = 12 total rats/time point. All values are means ± s.e.m. **P < 0.03.

We next studied the acute changes induced by mitochondria transplantation, which may instigate their long-term effects. We studied markers of three intevowen processes, mitochondria function, immune response, and neuronal activity. First, we followed entrance efficiency of MitoTracker-orange labeled mitochondria into mPFC neurons, astrocytes and microglia (Fig. 3a–d). Three hours post-transplantation, labeled mitochondria preferentially entered neurons both in Poly I:C and saline rats. Few mitochondria were identified inside astrocytic processes, and almost none in microglia. The latter might be due to either mitochondria disability to enter or their instant degradation by microglia. There was no significant difference in labeled-mitochondria entrance into neurons between PM and SM (300.8 ± 24.4 and 267.7 ± 52.6 intensity/volume).

Mitochondria entrance into neurons was associated with increased expression of c-Fos, an early functional marker for neural activation [49], in PM as compared to PV rats (PM vs. PV, *P* < 0.026; PV vs. SV, *P* < 0.031), reaching SV levels two days after transplantation (Fig. 3e, f). However, in SM rats, c-Fos was not affected. Seven days after transplantation c-Fos activation was similar in all groups (Supplementary Fig. 2a).

**Fig. 4:**
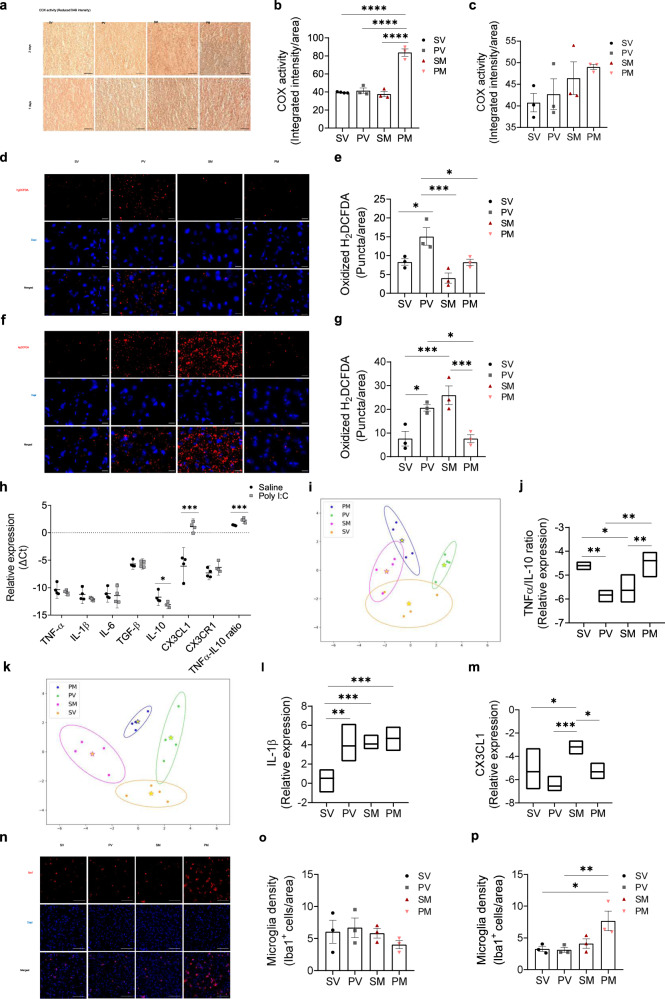
Acute effects of mitochondria transplantation on mitochondrial function and immune response. **a** Representative images of reduced 3,3’-diaminobenzidine (DAB), the electron acceptor of COX, in the mPFC of the four experimental groups at two and seven days after transplantation. Darker brown color of reduced DAB represents increased ex-vivo activity of COX. Scale bar: 50 µm. **b**, **c** Quantification of COX activity at two (**b**) and seven (**c**) days after transplantation. **b** One-way ANOVA showed significant differences in COX activity between groups two days post-procedure (*F*(3,9) = 66.4, *P* < 0.0001). Mitochondria transplantation significantly increased the activity of COX in PM as compared to all other groups (*P* < 0.0001). **c** Seven days after surgery, COX activity was similar in all groups (*F*(3,8) = 1.17, *P* = 0.241). *N* = 3 animals/group; 6–8 sections/animal; *n* = 12 total animals/time point. **d** **f** Representative images of oxidized H2DFCDA (red) of the four experimental groups at two (**d**) and seven (**f**) days after transplantation. Scale bar: 20 µm. **e**, **g** Quantification of ROS production at two (**e**) and seven (**g**) days after transplantation. **e** Two days after transplantation, one-way ANOVA showed a significant difference between groups in ROS production (*F*(3,8) = 9.54, *P* < 0.005). ROS production was significantly increased in PV as compared to all other groups, while PM ROS level was reduced to that of SV (PV vs. SV, *P* < 0.048; PV vs. PM, *P* < 0.045; PV vs. SM *P* < 0.003; PM vs. SV, *P* = 0.999). Mitochondrial transplantation had no significant effect in SM rats (SM vs. SV, *P* = 0.246). **g** Seven days after transplantation, one-way ANOVA showed a significant difference between groups in ROS production (*F*(3,8) = 11.6, *P* < 0.003). Sustained significant increase in PV as compared to SV (*P* < 0.039) and normal levels in PM are depicted (PM vs. PV, *P* < 0.038 and PM vs. SV, *P* > 0.999). However, in the SM group, a substantial increase was observed as compared to SV and PM groups (*P* < 0.006). *N* = 3 animals/group; 6 sections/animal; *n* = 12 total animals/time point. **h**–**m** Transcripts of the pro-inflammatory TNF-α, IL-1β, IL-6, the anti-inflammatory IL-10, TGF-β cytokines, the chemokine CX3CL1 and its receptor CX3CR1 and the inflammatory homeostasis index TNF-α/IL-10 ratio were measured by real-time PCR in the mPFC of two control (Poly I:C and saline offspring) and four experimental groups. **h** In Poly I:C offspring, a significant decrease was observed in IL-10 (*P* < 0.043) and an increase in CX3CL1 (*P* < 0.0008) and TNF-α/IL-10 ratio (*P* < 0.003) transcript levels as compared to saline rats. **i**, **k** Linear discriminant analysis (LDA) of groups’ immune profiles based on the expression of the immune factors (ΔΔCt values) at two (**i**) and seven (**k**) days following mitochondria transplantation. For each group, the ellipse represents ±2σ around the group score centroid. **i** A significant canonical discrimination between the immune profiles of the four experimental groups (*P*_*F1*_ < 0.031) shows an overlap between groups two days after the surgical procedure. **j** One-way ANOVA showed that the significant discriminating predictor at two days, TNF-α/IL-10 ratio (Wilk’s λ = 0.518; 93.8%), was similar between PM and SV groups (*P* > 0.05), and between PV and SM groups. The latter groups were significantly lower from both SV (*P* < 0.021 and *P* < 0.035, respectively) and PM (*P* < 0.012 and *P* < 0.02, respectively). **k** Seven days after transplantation, a significant canonical discrimination between immune profiles of the four experimental groups (*P*_*F1*_ < 0.002 and *P*_*F2*_ < 0.015) shows relatively disparate groups. **l**, **m** Seven days after transplantation, one-way ANOVA showed two significant discriminating predictors IL-1β (Wilk’s λ = 0.675; 100%) (**l**) which was significantly increased in all groups as compared to SV (at least *P* < 0.010), and CX3CL1 (Wilk’s λ = 0.554; 100%) (**m**), which was significantly increased in SM compared to all other groups (at least *P* < 0.04). Values are means of ΔCt (**h**) and ΔΔCt (**j**, **l**, **m**) ±95% confidence interval. **h**–**m** *N* = 4 animals/group, assessed in triplicates. **n** Representative images of immunofluorescence staining of Iba1, a microglial-specific calcium-binding protein, in the mPFC of the four experimental groups seven days after transplantation. **o**, **p** Quantification of Iba1+ cells at two and seven days after transplantation. Iba1+ cells were quantified as the sum of Iba1 staining (red) co-localized with Dapi (blue) divided by the tissue area. **o** Two days after transplantation, no differences were observed between groups (*F*(3, 8) = 0.799, *P* = 0.528). **p** Seven days after transplantation, significant differences were observed between groups in the number of Iba1+ microglia in the mPFC (*F*(3,8) = 5.56, *P* < 0.023) due to an increase in microglia number in PM as compared to SV and PV (PM vs. SV, *P* < 0.036; PM vs. PV, *P* < 0.030). *N* = 3 animals/group; 4–5 sections/animal; *n* = 12 total animals/time point. Scale bar: 100 µm. All values are means ± s.e.m. *P < 0.05; **P < 0.03; ***P < 0.006; ****P < 0.0001.

Next, we studied mPFC ex-vivo activity of COX (complex IV) of the electron-transfer-chain (ETC), and of SDH, participating both in mitochondrial tri-citric acid cycle and in ETC (complex II) (Fig. 4a–c and Supplementary Fig. 2b–d) both implicated in SZ [50, 51]. COX activity was significantly increased two days after transplantation in PM as compared to all other groups (*P* < 0.0001) with no significant difference between groups seven days later. SDH activity was not affected at both time-points. ROS, which are implicated in SZ [52, 53], are primarily produced by mitochondria, considered as signal mediators, and involved in cell growth, differentiation, progression and death [54, 55]. Consistent with the literature [2], an increase in the ROS indicator, oxidized H_2_DCFDA, was evidenced in PV as compared to the SV group two and seven days following surgery (*P* < 0.048 and *P* < 0.039, respectively) (Fig. 4d–g). Mitochondria transplantation significantly decreased ROS in PM rats as compared to PV (*P* < 0.045) restoring it to normal levels already at two days, which lasted for at least seven days following transplantation (PM vs. PV, *P* < 0.038). In the SM group, however, ROS massively increased seven days after surgery (SM vs. SV or PM, *P* < 0.006).

There is a well-established interplay between mitochondria and the immune system. We therefore studied the effect of transplanted mitochondria on mPFC inflammatory response. We assessed transcripts’ level of pro-inflammatory TNF-α, IL-1β, IL-6, anti-inflammatory IL-10, TGF-β cytokines, the chemokine CX3CL1 and its receptor CX3CR1, both indicators of neuron-microglia crosstalk, and calculated TNF-α/IL-10 ratio, an index of immune homeostasis (Supplementary Fig. 2e, f). First, we assessed cytokine transcripts in naïve Poly I:C and saline offspring rats in adolescence. We observed a significant reduction in IL-10 (*P* < 0.043), an increase in CX3CL1 (*P* < 0.0008) and a significantly higher TNF-α/IL-10 ratio in Poly I:C as compared to saline offspring (*P* < 0.003) (Fig. 4h), indicating an imbalance between major pro- to anti-inflammatory cytokines in Poly I:C offspring. Following transplantation, significant canonical discrimination of the immune profile between groups was obtained by LDA at two and seven days post-procedure (2 days: *P*_*F1*_ < 0.031, 88% between-group variability; 7 days: *P*_*F1*_ < 0.002 and *P*_*F2*_ < 0.015 with 86.7% and 81.9% between-group variability for all predictors, respectively) (Fig. 4i, k). At two days, all groups’ immune profile centroids±2σ were more converged than at seven days, suggesting predominance of surgical procedure in the immune response at two days, which declined at the later time point. Further analysis of the structure matrix revealed TNF-α/IL-10 ratio (Wilk’s *λ* = 0.518) as the significant predictor with 93.8% corrected group classification at two days (Fig. 4j), and IL-1β (Wilk’s λ = 0.675) and CX3CL1 (Wilk’s λ = 0.554) with 100% corrected group classification at seven days after transplantation (Fig. 4l, m). TNF-α/IL-10 ratio in PM group, reached SV values, while those of PV and SM were significantly lower from both SV (*P* < 0.021 and *P* < 0.035, respectively) and PM (*P* < 0.012 and *P* < 0.02, respectively), suggesting impaired immune response balance in PV and SM rats, which was restored in PM rats. IL-1β was significantly increased in all groups as compared to SV (at least *P* < 0.01), yet CX3CL1 was significantly increased in SM as compared to all other groups (at least *P* < 0.041), suggesting neuronal immune-stress in SM group is similar to that observed in the naïve Poly I:C group. Concomitantly, Iba1^+^ microglia, the CNS immune-residents, showed infiltration into the mPFC compared to their density in adjacent M1 cortical area in all groups two days after surgery (paired Student’s *t*-test, *P* < at least 0.04) (Fig. 4n–p and Supplementary Fig. 2g, h). Iba1^+^ microglia declined at seven days in all groups except for the PM group as compared to SV (*P* < 0.036). Interestingly, Iba1^+^ microglia density was negatively correlated with TGF-β (*r* = −0.973, *P* < 0.03) at seven days, while at two days with TNF-α/IL-10 ratio (*r* = −0.913, *P* < 0.05) in the PM group.

### The inverse effect of mitochondria transplantation in hLCLs

**Fig. 5:**
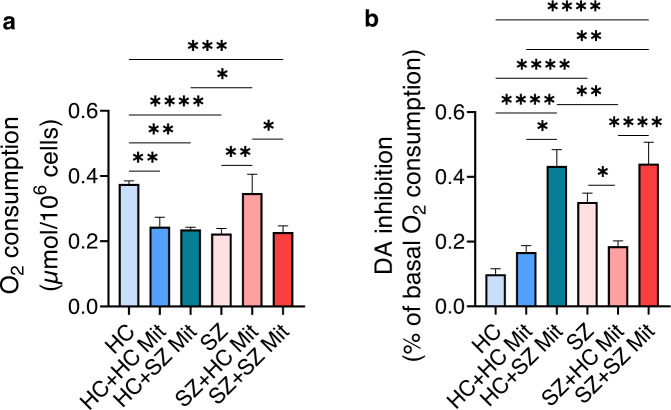
Mitochondria transplantation into hLCLs affects their basal cellular respiration and its inhibition by DA, depending on disease/health conditions. Isolated active mitochondria derived from either healthy controls (HC) or SZ patients were transplanted into HC- and SZ-hLCLs and their O_2_ consumption, as well as its inhibition by DA, were measured by Clark oxygen electrode five days after delivery. **a** Significant differences in basal O_2_ consumption between the six experimental groups (*F*(5,34) = 7.67, *P* < 0.0001). Reduced respiration of SZ-hLCLs (SZ vs. HC, *P* < 0.0003) was restored to normal levels after transplantation with mitochondria derived from HC (SZ vs. SZ + HC-Mit, *P* < 0.0085; HC vs. SZ + HC-Mit, *P* > 0.05) but remained impaired after transplantation with mitochondria isolated from SZ-hLCLs (SZ vs. SZ + SZ-Mit, *P* > 0.05; HC vs. SZ + SZ-Mit, *P* < 0.0012;). Mitochondria transplantation into HC-hLCLs impaired basal respiration regardless of mitochondria origin (HC vs. HC + HC-Mit, *P* < 0.0071; HC vs. HC + SZ-Mit, *P* < 0.0025). **b** One-way ANOVA shows significant differences in DA inhibition of O_2_ consumption between groups (*F*(5,36) = 15.9, *P* < 0.0001). Inhibition of O_2_ consumption by DA was increased in SZ-hLCLs as compared to HC-hLCLs (HC vs. SZ, *P* < 0.0001) and was attenuated to normal levels by transplanted HC-mitochondria (SZ vs. SZ + HC-Mit, *P* < 0.049; SZ + HC-Mit, vs. HC *P* > 0.05) but not by SZ-mitochondria (HC vs. SZ + SZ-Mit, *P* < 0.0001; SZ vs. SZ + SZ-Mit, *P* > 0.05). SZ-mitochondria severely increased DA inhibition in HC-hLCLs (HC vs. HC+SZ-Mit, *P* < 0.0001), while HC-mitochondria had no effect on DA induced inhibition of O_2_ consumption (HC vs. HC + HC-Mit, *P* > 0.05). *N* = 4–11 cell lines/group, measured in triplicates. All values are means ± s.e.m. *P < 0.05; **P < 0.01; ***P < 0.001; ****P < 0.0005.

Previously we have shown that mitochondrial respiration, as well as the extent of its inhibition by DA, are impaired in SZ-derived cells, and transplantation of HC-derived mitochondria restored both to normal levels [15, 25]. These findings were repeated using different hLCLs (basal respiration and its inhibition by DA, *P* < 0.009 and *P* < 0.049, SZ-hLCLs+HC-Mit vs. SZ-hLCLs, respectively) (Fig. 5a, b). However, transplantation of HC-derived mitochondria into HC-hLCLs significantly reduced basal respiration to SZ-hLCLs levels (HC-hLCLs+HC-Mit vs. HC-hLCLs, *P* < 0.007). Transplantation of SZ-derived mitochondria impaired both parameters in HC-hLCLs mimicking SZ-hLCLs pattern (basal respiration and its inhibition by DA, HC-hLCLs+SZ-Mit vs. HC-hLCLs, *P* < 0.002 and *P* < 0.0001, respectively), but did not further impair SZ-hLCLs. These data substantiate cells- and mitochondria origin-dependent beneficial/detrimental effects in hLCLs, which have been repeatedly used as a “neuronal proxy” to explore biological mechanisms of psychiatric disorders.

### Discussion

The present data demonstrate that mitochondria are crucial factors in determining the trajectory of late brain development and thereby behavior. We showed that a single transplantation of isolated healthy mitochondria in adolescence into the rat mPFC led to an opposite pattern of change in behavior and neuronal function in adulthood depending on host state, namely beneficial in the neurodevelopmental MIA model of SZ, yet detrimental in normal rats. Such an opposite pattern initiated shortly after transplantation, during which exogenous mitochondria interfered with mitochondrial COX activity, ROS and the immune response. The similar pattern of change in adolescence and adulthood in both Poly I:C and saline offspring suggests that interference with host-state during late developmental stages of the prefrontal cortex contributes to the long-term consequences.

Long-term opposite outcomes of mitochondria transplantation were manifested at the level of mPFC-regulated behavior, neuronal structure and transmission and intracellular processes. Our previous study showed that mitochondrial state was affected, as mitochondrial Δψm, the driving force for ATP production, was restored to normal in Poly I:C but impaired in control adult rats by the transplantation [15].The effect of mPFC mitochondria transplantation on behavior was probably restricted to the region of intervention as behaviors involving activation of the mPFC, latent inhibition [15] and locomotor agitation in a novel environment, were altered, while social recognition and amphetamine-induced activity, for which activation of the orbitofrontal cortex and striatum, respectively, is critical [37, 38], were not affected. Behavioral improvements in PM were associated with enhanced monoamines’ transmission solely in the mPFC, as well as normalization of pyramidal neurite outgrowth and dendritic spine density. However, in SM rats, the deleterious behavioral outcome was associated with no change in monoamines’ transmission, and impaired neuronal sprouting. Proteomics and phosphoproteomics provided several insights regarding the intracellular pathways that possibly underlie the substantial recovery and deterioration in PM and SM, respectively. Considering the limited knowledge regarding the downstream outcome of numerous phosphorylation sites and that most of the relevant tools cannot take into account multiple phosphorylated sites of a given protein simultaneously, the information that could be driven from phosphoproteomics is partial. Regardless, by various types of data analyses, we detected differences between groups that predominantly converged into two intracellular pathways, neuronal multifaceted functions and morphology, and energy metabolism. WGCNA showed a close link between the significantly differentiated metabolic and neuronal processes. The PM proteome and phosphoproteome profiles showed a considerable similarity to SV rats with by-and-large activation (vs. PV) of multiple intracellular pathways regulating neuronal activity and vitality (e.g. synaptogenesis, LTP and ERK/MAPK signaling pathways) and metabolic processes (e.g. carbohydrate glucose homeostasis, fatty acid metabolism and mitochondrial processes). However, the SM group showed a resemblance to PV rats, mainly observed by its shift towards PV in proteome profile and in the phosphoproteome core functions enrichment, including neuronal processes and energy metabolism. In all, the altered proteome profiles possibly underlie the upstream improvements or impairments in neuronal function and behavior. Indeed, a large body of proteomic evidence in SZ has identified consistent disruption mainly in neuronal transmission, synaptic plasticity, neurites outgrowth, cytoskeleton arrangement, calcium signaling, oxidative stress and energy metabolism [6, 56, 57].

The long-term change was evoked by a preferential entrance of exogenous mitochondria into neurons, equally into both Poly I:C and saline rats. The latter is supported by our findings in SZ and HC-derived hLCLs, in which isolated mitochondria enter cells with the same efficiency regardless of donor or host origins (unpublished data). It is not clear why mitochondria preferentially entered neurons rather than astrocytes or microglia, except for neurons’ exceptional high-energy demands. Indeed, neurons in need incorporate functional mitochondria released by astrocytes [54].

Mitochondria are essential for neuronal proper functioning yet also for oxidative stress-mediated cellular damage, with ROS playing an important role in both. Notably, it has been reported that an altered redox state is involved in the regulation of various transcription factors and protein activators thereby modulating signaling pathways as well as dendritic length and spine density in neurons [55, 58]. In addition, ample evidence shows that mitochondria, their mtDNA and ROS are heavily involved in inflammation induction [59]. On the other hand, the production of pro-inflammatory mediators, such as TNF-α and IL-1β, impairs mitochondrial function by increasing ROS and decreasing respiratory complexes activity and ATP production [60]. These interacting processes, all implicated in SZ [4, 6, 52, 61], were differentially modulated in PM and SM rats shortly after mitochondrial transplantation. In PM rats, mitochondria transplantation induced an early activation of neurons (assessed by c-Fos), improvement in mitochondrial functions indicated by normalization of ROS production and a temporary increase in COX activity as well as restoration of the immune balance (expressed by TNF-α/IL10 ratio). In SM rats, however, transplantation had no effect on neuronal early activation, caused a massive increase in ROS, induced an early immune imbalance and a later increase in the neuronal secreted stress chemokine CX3CL1. Interestingly, only in PM rats, mPFC microglia increased density persisted seven days after surgery, whilst cytokines’ levels were attenuated, suggesting the involvement of microglia in synaptic re-organization, facilitating synaptic plasticity and remodeling neuronal network [62]. The mechanism by which mitochondria induce long-term effects is still elusive. It is reasonable to assume that exogenous mitochondria do not last long in brain, as the majority of mitochondrial proteins are encoded by the nDNA and endogenous mitochondria turnover rate in brain is maximally 25 days [63]. It is also not likely that mtDNA is responsible for the long-term effects as our in-vitro findings showed that donor mitochondrial DNA could not be detected in host cells 10 days after transplantation, whereas their effect on respiration lasted for >28 days [15] (and unpublished data). Regardless, our study shows that the outcome of interference is highly dependent on host physiological conditions such as the immune system, oxidative state and bioenergetics.

This study suggests a causal link between mitochondria inherent state and adolescent brain development. Still, several questions remain open. For example, the importance of transplantation timing for the long-term outcomes; which inherent processes mediate mitochondrial transplantation-induced effects; how mitochondria-instigated acute effects lead to the long-term consequences; how exogenous mitochondria interfere with host-specific mitochondrial homeostasis; and how/whether mitochondrial transplantation-induced changes in the mPFC project to additional PFC subregions or to other brain regions implicated in late stages of brain development and/or in SZ. In this study, CoI activity was not assessed despite its major role in the pathophysiology of SZ [4, 50]. To the best of our knowledge there is no histochemical assay for CoI, probably since its substrate NADH is metabolized by additional cellular dehydrogenases. The reliable and reproducible enzymatic or in-gel activity assessments in isolated mitochondria [26, 64] were also not applicable due to the mPFC size. Finally, the relevance to SZ of the host-dependent opposite effects of transplantation in rats was demonstrated in SZ and healthy subjects-derived hLCLs, which showed donor- and host- dependent inverse shift in mitochondrial function due to the interference with their original state. hLCLs have been shown to retain many SZ-related abnormalities including those of mitochondria and are suggested as a medication-free peripheral cell model for the disorder. Further studies are warranted to explore the mechanism of interaction between transplanted and host mitochondria depending on their health/disease origin in CNS-relevant culture models. Regardless, the present study supports the potential of mitochondria manipulation as a novel treatment for disorders with neurodevelopmental and bioenergetic deficits, such as SZ. In addition, it emphasizes the need to consider possible detrimental effects induced by the two-edge sword nature of mitochondria and their possible interaction with subject’s physiological status, pharmacotherapy, immunological and mitochondrial inherent state. In this respect, it is notable that beneficial/detrimental effects of mitochondrial transplantation in SZ/healthy subjects-derived hLCLs were depended on both donor mitochondria origin and host mitochondrial state.

Supplementary information is available at MP’s website.

## Supplementary information

Supplementary information

Supplementary material

Supplementary material
