# Supplementary material for: Mitochondria play an essential role in the trajectory of adolescent neurodevelopment and behavior in adulthood: evidence from a schizophrenia rat model
Source: Mol Psychiatry. 2022 Nov 15;28(3):1170–81. doi: 10.1038/s41380-022-01865-4 (PMC10005953; doi:10.1038/s41380-022-01865-4)
Supplement: Supplementary file 1 — Supplementary information [file 41380_2022_1865_MOESM1_ESM.docx]

Supplementary information

Supplementary Methods

Maternal immune activation (MIA) and human LCLs models of SZ

On gestational day 15, pregnant Wistar rat dams were administered with Poly I:C (4 mg/ml/kg; Sigma-Aldrich, Israel) or saline injection into the tail vein under 4% isoflurane anesthesia. On postnatal day (PND) 28, Poly I:C and saline offspring were assigned to two experimental groups (Poly I:C or saline exposed groups), each consisted of male and female offspring derived from multiple independent litters. On PND 34, Poly I:C and saline offspring were assigned to intracerebral mitochondria or vehicle transplantation. Four experimental groups were obtained: (1) saline and (2) Poly I:C prenatally exposed offspring injected with vehicle (SV and PV, respectively), (3) saline and (4) Poly I:C prenatally exposed offspring transplanted with mitochondria (SM and PM, respectively).

Mitochondria transplantation

Adolescent Poly I:C and saline offspring were anesthetized intraperitoneally with Ketamine/Xylazine cocktail (100/20 mg/kg), injected with analgesic Buprenorphine (0.05 mg/kg), and fixed in a stereotaxic frame (RWD Life Science, USA). Freshly prepared isolated active mitochondria (100 µg/4.5 µl) or vehicle (final suspension buffer) were injected bilaterally into the mPFC, defined as containing the following sub-regions; cingulate gyrus 1 (CG1; AP+2.3, ML±0.7, DV-2.5), prelimbic (PrL; AP+2.3, ML±0.7, DV-3.5) and infralimbic (IL; AP+2.3, ML±0.7, DV-4.9) cortices all implicated in SZ^2,4,23,24,52^. Each rat received a single bilateral stepwise injection using a customized 30G needle (Ophir Analytics, Israel), with 33.33 µg/15 µl/3 min starting from the IL cortex.

In all, 150 animals were used for all analyses in four independent experiments. For behavioral testing and Golgi-cox staining, N=50; for proteomics and HPLC, N=48; for immunostaining, ROS and immune factors’ expression, N=40, 4 groups/each parameter. For mitochondria entrance into brain cells assessment, N=4-6; for immune factors’ expression of naïve offspring N=8, 2 groups/each parameter.

Behavioral studies

*Social recognition:* The test was conducted in an apparatus divided into three equal-sized compartments (one middle and two lateral) by black Plexiglas partitions containing two transparent plastic cups with holes placed on either lateral sides of the apparatus. Before testing day, test rats were habituated to the apparatus for two consecutive days for 20 min, free to explore the middle chamber. On test day, the test rat was placed into the middle compartment for initial habituation of 10 min during that time the two partitions covered the lateral compartments. Thereafter, the partitions were removed and the familiar (a littermate) male or female rat (the familiar and test rats were of the same sex) was placed in one of the cups (familiar stimulus). The other cup remained empty (non-familiar stimulus).

Golgi-Cox staining

Following a 21 day processing period, tissues were sliced coronally (100 µm), dehydrated and mounted on gelatin-coated slides with Eukitt mounting (Sigma-Aldrich, Israel). A 1 µm interval z-series were captured using a 3DHistech Pannoramic 250 Flash III slide scanner with a Plan-Apochromat x40 objective connected to an Adimec Q12A180 camera. Digital zoom was available up to x165 using the CaseViewer software.

*Dendrite reconstruction:* To obtain accurate measurements of dendritic morphology, strict inclusion criteria were used for the selection of the impregnated neurons before quantitative analysis as described previously^57^. Briefly, only neurons lying within layer II/III of the mPFC sub-regions CG1, PrL or IL cortices, exhibiting complete filling of intact dendritic arbors and displaying pyramidal cell morphology were included in the analysis. Dendrites were reconstructed using the semi-manual NeuronJ plugin for Fiji software (<https://imagescience.org/meijering/software/neuronj/>), classified to primary, secondary, tertiary, quaternary and quinary order and quantified. Six to ten neurons/animal, 4-5 animals/group were analyzed.

*Spine density:* To count dendritic spines, straight branches of secondary, tertiary and quaternary dendrites that provided clear resolution of spines were selected, and spine density was calculated as the number of spines per 10 μm of dendrite for nine segments per cell and five cells per animal (n=4-5 animals/group). Spines were quantified using an in-house macro Fiji script and validated manually with 95% accuracy.

Immunofluorescence

*Antibodies:* Primary antibodies: NeuN (1:500, mouse, Millipore, MAB377), Iba1 (1:250, rabbit, WAKO, 019-19741), GFAP (1:500, chicken, Abcam, ab4674), and c-Fos (1:2000, rabbit, Abcam, ab190289 ). Secondary antibodies: AlexaFlour 488 (1:400, donkey anti-rabbit, Abcam, ab150073 ), AlexaFlour 488 (1:500, donkey anti-mouse, Abccam, ab150105), AlexaFlour 594 (1:250, donkey anti-rabbit, Abcam, ab150076 ), AlexaFlour 647 (1:400, donkey anti-chicken, Enco, 703-605-155).

Image data analysis

Image analysis was conducted on c-Fos, SDH, COX, H_2_DCFDA and Iba1 data by the Image Processing Unit of the BCF, Faculty of Medicine, Technion, using dedicated codes written in Python (version 3.7.9) and based on relevant images and data analysis libraries. e.g., numpy, scipy, tensorflow, pandas, skimage and stardist. Image analyses were run on a PC with 64 Gb ram and Nvidia RTX 2060 graphics card. The pipeline for each image was similar, including reading and separation to channels. Then, each channel was duplicated and converted into a binary image. A threshold was applied on the binary image (usually based on an Otsu threshold) in order to produce a global mask image. This mask was applied back on the original channel image for separating the tissue (mPFC) area from its background. In order to segment nuclei or fluorescent expression, different segmentation methods were implemented. In case of nuclei, representing convex entities, we implemented a pre-trained CNNs (convolutional neural networks). In other cases, we used color segmentation, followed by image refinement made by removing small and large objects. For co-localization type of analyses, another image was produced depicting the overlap of nuclei and fluorescent expression, and then was further used in the image and data analysis. All data analyses used data frames. These enabled storing relevant information, starting from experimental data, to other valuable data retrieved from the image analysis stage. The latter, included labels, areas, mean intensities, nuclei coordinates, etc. Data analysis stage allowed further data filtering and calculations. The Python codes for data analyses are available from the corresponding author upon reasonable request.

Mitochondria functional parameters

*Succinate dehydrogenase (SDH) and Cytochrome C oxidase (COX) histochemistry:* Briefly, for SDH activity, frozen brain sections were incubated for 20 min at 37^◦^C with its substrate 50 mM succinic acid, the electron acceptor 1.5 mM nitro blue tetrazolium (NBT) and 1mM potassium cyanide (KCN) in the presence or absence of SDH inhibitor 0.01 M sodium malonate. For COX activity, frozen brain sections were incubated for 1.5 h at 37^◦^C with its substrate 0.1% reduced cytochrome *c* and its electron acceptor 0.1% 3,3’-diaminobenzidine (DAB) in the presence or absence of COX inhibitor 0.01 M KCN. Following incubation, all slides were washed and mounted with a warm (60^◦^C) glycerol-gelatin medium (Sigma-Aldrich, Israel).

*Reactive oxygen species (ROS) production:* mPFC frozen brain sections were washed in PBS at room temperature, incubated with 25 µM H_2_DCFDA for 30 min, washed three times with PBS and mounted with DAPI Fluoromount-G (SouthernBiotech). Oxidized H_2_DCFDA (excitation 450-490 nm, emission 500-550 nm) and DAPI (excitation 335-383 nm, emission 420-470 nm) were assessed.

*Mitochondrial basal respiration and its inhibition by DA in hLCLs:* Oxygen consumption was measured polarographically with a thermostatically controlled (37^o^C) Clark oxygen electrode (Strathkelvin 782 Oxygen System, North Lanarkshire, Scotland). CoI driven respiration was assessed in digitonin (0.001%) permeabilized HC and SZ-derived hLCLs transplanted with mitochondria or vehicle (2-5x10^6^/assessment), by the addition of the CoI substrates glutamate (5 mM) and malate (1 mM). Inhibition by DA (10^-4^M) was assessed following pretreatment with tranylcypromine (10^-5^M, monoamine oxidase inhibitor) for 30 min.

Proteomics and phosphoproteomics

*Protein extraction and trypsinization:* mPFC samples (n=4/group; 4 groups) were homogenized in lysis buffer (8 M urea, 75 mM NaCl, 50 mM Tris pH=8.2, Protease inhibitor, 1 mM NaF, 1 mM β-glycerophosphate, 1 mM Sodium Orthovandate, 10 mM Sodium Pyrophosphate, 1 mM PMSF), and sonicated (90%, 10-10, 5'). Cysteines were reduced with 2.8 mM DTT (60ºC for 30 min) and modified with 8.8 mM iodoacetamide in 100 mM ammonium bicarbonate (in the dark, room temperature for 30 min). The proteins were digested in 1 M Urea with modified trypsin (Promega) at a 1:50 enzyme-to-substrate ratio, overnight at 37^o^C. Additional second trypsinization was done for 4 hrs.

*Phosphopeptides enrichments:* The tryptic peptides were desalted using C18 tips (Sepak, Waters) partially dried, re-suspended in 40% Acetonitrile (ACN), 6% Trifluoroacetic acid (TFA), and enriched for phosphopeptides on titanium dioxide (TiO2) beads. Titanium beads were pre-washed (80% ACN, 6% TFA) mix with the peptides for 10 min at 37ºC, washed with 30% ACN with 3% TFA and 80% ACN with 0.1% TFA. Bound peptides were eluted with 20% ACN with 325 mM Ammonium Hydroxide followed by 80% ACN with 325 mM Ammonium Hydroxide. The resulted peptides were desalted using C18 tips and analyzed by LC-MS/MS.

*Mass Spectrometry:* The resulted peptides were analyzed by LC-MS/MS using a Q- Exactive Plus mass spectrometer (Thermo) fitted with a capillary HPLC (easy nLC 1000, Thermo-Fisher). The peptides were loaded onto a C18 trap column (0.3 x 5 mm, LC-Packings) connected online to a homemade capillary column (20 cm, 75 micron ID) packed with Reprosil C18-Aqua (Dr. Maisch, GmbH, Germany) in solvent A (0.1% formic acid in water). The peptides mixture was resolved with a (5 to 28%) linear gradient of solvent B (95% acetonitrile with 0.1% formic acid) for 180 min followed by a 15 min gradient of 28 to 95% and 25 min of 95% acetonitrile with 0.1% formic acid in water at flow rates of 0.15 μl/min. Mass spectrometry was performed in a positive ion mode (at a mass range of m/z 350–1800 AMU and resolution 70,000) using repetitively full MS scan followed by collision induces dissociation (HCD, at 35 normalized collision energy) of the 10 most dominant ions (>1 charges) selected from the first full MS scan.

*Mass Spectrometry Data Analysis:* The mass spectrometry data were analyzed using the MaxQuant software 1.5.2.8. (<https://www.maxquant.org>) for peak picking identification and quantitation using the Andromeda search engine^60^, searching against the Rat proteome from the Uniprot database with a mass tolerance of 20 ppm for the precursor masses and 20 ppm for the fragment ions. Methionine oxidation, phosphorylation (STY) and protein N-terminus acetylation were accepted as variable modifications and carbamidomethyl on cysteine was accepted as static modification. Minimal peptide length was set to six amino acids and a maximum of two miscleavages was allowed. Peptide- and protein-level false discovery rates (FDRs) were filtered to 0.01 using the target-decoy strategy. The protein table was filtered to eliminate the identifications from the reverse database and common contaminants. The data were quantified by label-free analysis using the MaxQuant software, based on extracted ion currents (XICs) of peptides enabling quantitation from each LC/MS run for each peptide identified in any of the experiments. Proteins and phosphoproteins were normalized after logarithmic transformation.

*Bioinformatics:* Unsupervised hierarchical clustering of Z-score-transformed ANOVA significant (P≤0.05) proteins and phosphoproteins was generated using Perseus followed by STRING analysis (<https://string-db.org/>) based on Gene Ontology (GO) database for biological processes, molecular functions and cellular components for dendrogram cluster analysis. Distances between samples were estimated using Euclidean distances. Based on previous multiple testing corrections methods in proteomics^36^, significantly altered proteins and phosphoproteins, which were changed by at least 25% (effect size cut-off of log2-ratio≥|0.32|), were further analyzed. ANOVA-significant proteins and phosphoproteins were analyzed for pathway enrichment by Over-Representation Analysis (ORA) using WebGestalt (<http://www.webgestalt.org/>). The bi-weight mid-correlations between significant carbon metabolic proteins and all-ANOVA significant phosphoproteins were analyzed using an R-package for Weighted Correlation Network Analysis (WGCNA) (<https://horvath.genetics.ucla.edu/html/CoexpressionNetwork/Rpackages/WGCNA/>), with proteins analyzed as traits and phospho-sites composing eigenprotein modules^37^. For differential pathway analysis, the following comparisons PV vs. SV, PM vs. PV, SM vs. SV and PM vs. SV were analyzed by Student’s t-test. Significant proteins and phosphoproteins, were analyzed using the Ingenuity Pathway Analysis (IPA) (<https://digitalinsights.qiagen.com/products-overview/discovery-insights-portfolio/analysis-and-visualization/qiagen-ipa/>) by core analysis to predict activation of canonical pathways. The significance of the association between the dataset and canonical pathway was measured by Z-score (prediction of activation=z-score >2; and inhibition=z-score <-2), and Fisher's exact test of the enriched pathways (significant at–log (P-value)>1.3). A gene-set enrichment analysis (GSEA) (<https://www.gsea-msigdb.org/gsea/index.jsp>)^38^ was performed to identify core functions of biological processes and pathways of the differentially expressed significant proteins or phosphoproteins in the above comparisons. Datasets were analyzed in reference to GO and KEGG databases on the chip platform Rat_UniProt_SwissProt_Human_Orthologs_MSigDB.v7.2.chip of the Molecular Signature Database (<http://www.broadinstitute.org/msigdb>). Data visualization of GSEA enrichments was performed in Cytoscape (<https://cytoscape.org/>) using the EnrichmentMap pipeline collection plugin (<https://apps.cytoscape.org/apps/enrichmentmap>). The direction of change was determined by Normalized Enrichment Scores (NES). Enrichment maps were created with the parameters of P≤0.05 and combined coefficient >0.375 with combined constant=0.5.


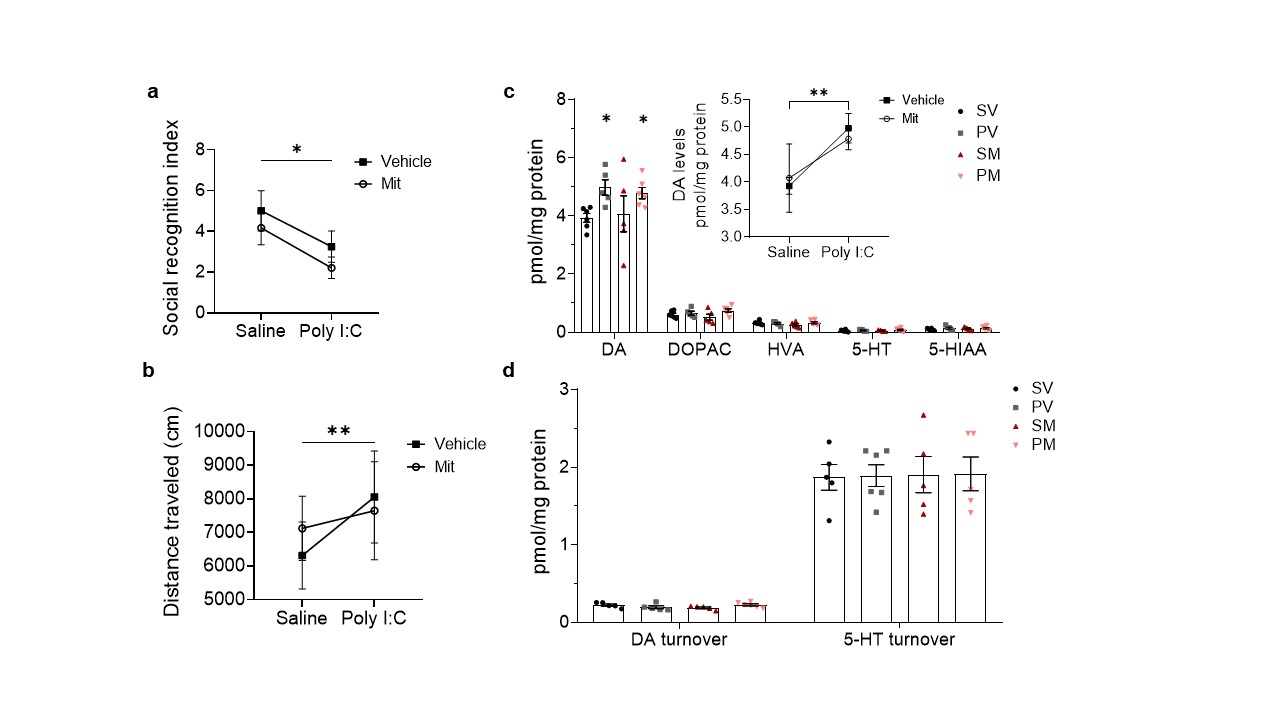
Supplementary Figures

Supplementary Fig. 1: Long-term effects of mitochondria transplantation on social recognition, amphetamine-induced activity, and monoamines’ levels in the striatum. Mitochondria transplantation showed no effect on SZ-related behaviors of social recognition and amphetamine-induced activity, orbito-frontal and striatal dependent, behaviors, respectively, in the adult offspring (PND>90). a, Both Poly I:C groups showed significantly lower social recognition index as compared to both saline groups (two-way ANOVA main effect: F(1,22)=4.48, P<0.045). b, Amphetamine-induced activity was significantly increased in both Poly I:C as compared to both saline groups (two-way ANOVA main effect: F(1,24)=6.839, P<0.015). N=5-8 animals/group. c, DA levels were significantly decreased in the striatum of both PV and PM groups as compared to both saline groups two-way ANOVA(main effect: F(1,18)=6.924, P<0.017). No significant differences were observed in monoamine and their metabolites and in d, DA and 5-HT turnover rates (P>0.05). N=5-8 animals/group. All values are means ± s.e.m. * P<0.05; ** P<0.02.


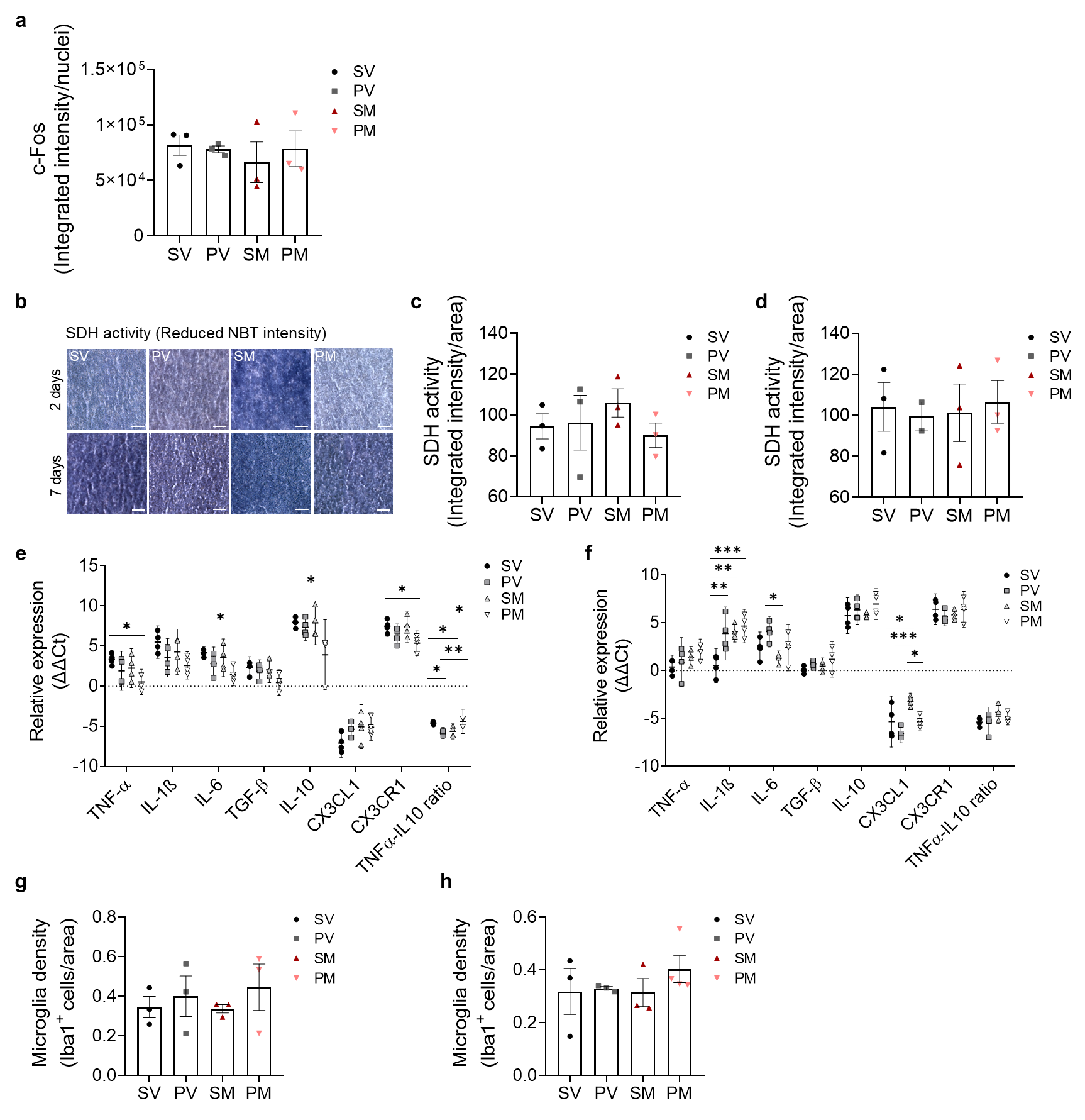


Supplementary Fig. 2: Acute effects of mitochondria transplantation on c-Fos activation and inflammatory response. a, No differences were observed between groups in c-Fos activation seven days post-procedure (F(3,8)=0.266, P=0.848). b, Representative images of reduced nitro blue tetrazolium (NBT), the electron acceptor of SDH, in the mPFC of the four experimental groups at two and seven days after transplantation. Darker blue color of reduced NBT represents increased ex-vivo activity of SDH. c, d, Quantification of SDH activity at two and seven days after transplantation. No significant differences were observed in SDH activity between all groups at both time points (F(3,8)=0.592, P=0.637 and F(3,7)=0.064, P=0.977, respectively). e, One way ANOVA shows significant differences in cytokine expression levels between experimental groups two days after transplantation. (TNF-α: (F(3,12)=3.539, P<0.048); IL-6: (F(3,12)=4.350, P<0.027); IL-10: (F(3,12)=4.976, P<0.018); CX3CR1: (F(3,12)=4.051, P<0.033) and TNF-α/IL-10 ratio: (F(3,10)=8.799, P<0.004)). Tukey’s post-hoc analysis, PM rats showed a significant reduction in TNF-α (P<0.032), IL-6 (P<0.02), IL-10 (P<0.028) and CX3CR1 (P<0.049) expression as compared to the SV group. TNF-α/IL-10 ratio was not different between PM and SV groups (P>0.05), while significantly lower in PV and SM as compared to SV (P<0.021 and P<0.035, respectively) and to PM (P<0.012 and P<0.02, respectively) groups. f, One way ANOVA shows significant differences in cytokine expression levels between experimental groups seven days after transplantation (IL-1β: (F(3,12)=9.315, P<0.002); IL6: (F(3,12)=4.490, P<0.025) and CX3CL1: (F(3,12)=8.194, P<0.003)). Tukey’s post-hoc analysis , PV, SM and PM groups showed a significant increase in IL-1β expression as compared to the SV group (P<0.01, P<0.007 and P<0.002, respectively). SM group showed elevated CX3CL1 expression levels as compared to all other groups (SM vs. SV, P<0.041; SM vs. PV, P<0.002; SM vs. PM, P<0.039). g, h, No differences between groups in Iba1+ microglia density in M1 cortical area at two and seven days post-procedure (One-way ANOVA: F(3,8)=0.374, P=0.774 and F(3,9)=0.613, P=0.623, respectively). a, c, d, g, h: N=3 animals/group; 6 sections/animal; N=12 total animals/time point. Values are means± s.e.m. e, f: N=4 animals/group, measured in triplicates. Values are ΔΔCt ± 95% confidence interval. * P<0.05; ** P<0.01; *** P<0.005.

Supplementary Tables

Supplementary Table 1: list of primer sequences

| Primer | Primer sequence | Product size (bp) |
| --- | --- | --- |
| Il-1β | F – CAGCTTTCGACAGTGAGGAGA  R – TGTCGAGATGCTGCTGTGAG | 138 |
| Il-6 | F – GCCCTTCAGGAACAGCTATGA  R – TGAAGTCTCCTCTCCGGACT | 123 |
| Tnfα | F – CTCAAAACTCGAGTGACAAGC  R – ACCACCAGTTGGTTGTCTTTG | 136 |
| Il-10 | F – GGGAGAGAAGCTGAAGACCC  R – TAGACACCTTTGTCTTGGAGCTTA | 128 |
| Tgfβ | F – AGTGGCTGAACCAAGGAGAC  R – CGTTTGGGACTGATCCCATTG | 112 |
| Cx3cl1 | F – GGCACAAAGTGTCTACTGAAGC  R – TTGGAGCTATTCAGAGCGGAAA | 134 |
| Cx3cr1 | F – GGACGCCTTACAACATCGTG  R – TAAACGCCACTGTCTCCGTC | 120 |
| Gapdh | F – TGCACCACCAACTGCTTAGC  R – GGCATGGACTGTGGTCATGAG | 70 |

Supplementary Table 2: Ingenuity Pathway Analysis (IPA) of proteomics

Significant canonical pathways of proteomics analyzed by IPA arranged by groups’ comparisons.

PV vs. SV comparison

| Ingenuity Canonical Pathways | -log (p-value) | Ratio | z-score | Molecules |
| --- | --- | --- | --- | --- |
| Sirtuin Signaling Pathway | 3.98 | 0.0309 | -2.121 | BAX,H1f4,IDH2,MAP1LC3A,MTOR,NDUFA8,NDUFS8,PDK1,SDHB |
| Synaptogenesis Signaling Pathway | 3.75 | 0.0288 | 0.378 | AP2B1,CACNB3,CPLX2,DNAJC5,HRAS,MTOR,PRKAR2A,RAC1,YKT6 |
| EGF Signaling | 3.38 | 0.0727 | -1 | CSNK2A1,CSNK2B,HRAS,MTOR |
| Netrin Signaling | 3.11 | 0.0615 | -2 | CACNB3,NCK2,PRKAR2A,RAC1 |
| Mitochondrial Dysfunction | 3.1 | 0.0351 | #NUM! | ACO1,COX17,GSR,NDUFA8,NDUFS8,SDHB |
| Gαi Signaling | 2.91 | 0.04 | 1 | CAV1,HRAS,PRKAR2A,RGS14,RGS7 |
| Semaphorin Neuronal Repulsive Signaling Pathway | 2.71 | 0.036 | 1.342 | DPYSL3,DPYSL4,GUCY1B1,PRKAR2A,RAC1 |
| mTOR Signaling | 2.64 | 0.0286 | -1.342 | EIF3M,HRAS,MTOR,PPP2R5A,RAC1,RPS6 |
| EIF2 Signaling | 2.5 | 0.0268 | #NUM! | EIF3M,EIF5,HNRNPA1,HRAS,RPL30,RPS6 |
| Regulation of eIF4 and p70S6K Signaling | 2.37 | 0.0301 | -1 | EIF3M,HRAS,MTOR,PPP2R5A,RPS6 |
| IGF-1 Signaling | 2.36 | 0.0385 | -2 | CSNK2A1,CSNK2B,HRAS,PRKAR2A |
| Paxillin Signaling | 2.3 | 0.037 | -2 | HRAS,NCK2,RAC1,TLN2 |
| Oxidative Phosphorylation | 2.28 | 0.0367 | 0 | COX17,NDUFA8,NDUFS8,SDHB |
| Methylglyoxal Degradation III | 2.19 | 0.105 | #NUM! | AKR1B1,AKR7A2 |
| Synaptic Long Term Depression | 2.14 | 0.0265 | -1.342 | CACNB3,GUCY1B1,HRAS,PLCL1,PPP2R5A |
| NRF2-mediated Oxidative Stress Response | 2.14 | 0.0265 | #NUM! | AKR7A2,DNAJC5,ERP29,GSR,HRAS |
| G Beta Gamma Signaling | 2.11 | 0.0328 | -2 | CACNB3,CAV1,HRAS,PRKAR2A |
| Clathrin-mediated Endocytosis Signaling | 2.1 | 0.0259 | #NUM! | AP2B1,CSNK2A1,CSNK2B,RAC1,SYNJ1 |
| ERK/MAPK Signaling | 2.02 | 0.0248 | -2.236 | HRAS,PPP2R5A,PRKAR2A,RAC1,TLN2 |
| TCA Cycle II (Eukaryotic) | 2 | 0.0833 | #NUM! | ACO1,SDHB |
| Ephrin B Signaling | 1.96 | 0.0417 | #NUM! | HRAS,NCK2,RAC1 |
| Integrin Signaling | 1.93 | 0.0235 | -2.236 | CAV1,HRAS,NCK2,RAC1,TLN2 |
| AMPK Signaling | 1.93 | 0.0235 | -1.342 | MTOR,PFKP,PPP2R5A,PRKAR2A,RAB22A |
| Insulin Receptor Signaling | 1.92 | 0.0288 | 1 | HRAS,MTOR,PRKAR2A,SYNJ1 |
| Proline Degradation | 1.72 | 0.333 | #NUM! | LOC102724788/PRODH |
| Dopamine-DARPP32 Feedback in cAMP Signaling | 1.69 | 0.0245 | 0 | GUCY1B1,PLCL1,PPP2R5A,PRKAR2A |
| GABA Receptor Signaling | 1.64 | 0.0316 | #NUM! | AP2B1,CACNB3,GABRB2 |
| Wnt/β-catenin Signaling | 1.61 | 0.0231 | -2 | CSNK2A1,CSNK2B,PIN1,PPP2R5A |
| PI3K/AKT Signaling | 1.52 | 0.0217 | 0 | HRAS,MTOR,PPP2R5A,SYNJ1 |
| PPARα/RXRα Activation | 1.47 | 0.0209 | #NUM! | CKAP5,HRAS,PLCL1,PRKAR2A |
| Synaptic Long Term Potentiation | 1.31 | 0.0233 | #NUM! | HRAS,PLCL1,PRKAR2A |

PM vs. PV comparison

| Ingenuity Canonical Pathways | -log(p-value) | Ratio | z-score | Molecules |
| --- | --- | --- | --- | --- |
| EIF2 Signaling | 5.85 | 0.0402 | -1 | HRAS,RPL10,RPL21,RPL24,RPL30,RPL7,RPLP1,RPS6,RRAS2 |
| Synaptogenesis Signaling Pathway | 4.69 | 0.0288 | 0.333 | ACTR2,AP2S1,ARPC3,EPHB2,HRAS,MTOR,RRAS2,STX16,WASF1 |
| Actin Cytoskeleton Signaling | 3.93 | 0.0308 | 1.633 | ACTR2,ARPC3,HRAS,KNG1,RRAS2,TLN2,WASF1 |
| Gluconeogenesis I | 3.58 | 0.115 | #NUM! | MDH2,ME3,PGK1 |
| Ephrin Receptor Signaling | 3.5 | 0.0317 | 2.236 | ACTR2,ARPC3,EPHB2,HRAS,JAK2,RRAS2 |
| Sirtuin Signaling Pathway | 3.27 | 0.0241 | 0.447 | ACLY,BAX,MTOR,NDUFA13,NDUFA8,PDHA1,PGK1 |
| Insulin Receptor Signaling | 3.24 | 0.036 | 1.342 | ACLY,HRAS,JAK2,MTOR,RRAS2 |
| Integrin Signaling | 3.23 | 0.0282 | 2.449 | ACTR2,ARPC3,CAV1,HRAS,RRAS2,TLN2 |
| Mitochondrial Dysfunction | 2.84 | 0.0292 | #NUM! | ACO1,COX17,NDUFA13,NDUFA8,PDHA1 |
| IGF-1 Signaling | 2.79 | 0.0385 | 2 | CSNK2A1,HRAS,JAK2,RRAS2 |
| Neuregulin Signaling | 2.77 | 0.0381 | 0 | HRAS,MTOR,RPS6,RRAS2 |
| EGF Signaling | 2.62 | 0.0545 | #NUM! | CSNK2A1,HRAS,MTOR |
| Clathrin-mediated Endocytosis Signaling | 2.61 | 0.0259 | #NUM! | ACTR2,AP2S1,ARPC3,CSNK2A1,EPHB2 |
| G Beta Gamma Signaling | 2.54 | 0.0328 | 2 | CACNA1E,CAV1,HRAS,RRAS2 |
| Reelin Signaling in Neurons | 2.54 | 0.0328 | 0 | ACTR2,ARPC3,MTOR,WASF1 |
| Ferroptosis Signaling Pathway | 2.49 | 0.0317 | 0 | HRAS,RRAS2,SLC3A2,SLC7A11 |
| p70S6K Signaling | 2.45 | 0.031 | 0 | HRAS,MTOR,RPS6,RRAS2 |
| Fatty Acid α-oxidation | 2.38 | 0.1 | #NUM! | ALDH1B1,ALDH3A2 |
| TCA Cycle II (Eukaryotic) | 2.23 | 0.0833 | #NUM! | ACO1,MDH2 |
| Insulin Secretion Signaling Pathway | 2.18 | 0.0205 | 0.447 | CACNA1E,JAK2,MTOR,PDHA1,STX16 |
| Glycolysis I | 2.16 | 0.0769 | #NUM! | PFKP,PGK1 |
| Regulation of eIF4 and p70S6K Signaling | 2.07 | 0.0241 | 0 | HRAS,MTOR,RPS6,RRAS2 |
| Dopamine Degradation | 2.04 | 0.0667 | #NUM! | ALDH1B1,ALDH3A2 |
| PI3K/AKT Signaling | 1.91 | 0.0217 | 1 | HRAS,JAK2,MTOR,RRAS2 |
| Paxillin Signaling | 1.81 | 0.0278 | #NUM! | HRAS,RRAS2,TLN2 |
| Oxidative Phosphorylation | 1.8 | 0.0275 | #NUM! | COX17,NDUFA13,NDUFA8 |
| NGF Signaling | 1.75 | 0.0263 | #NUM! | BAX,HRAS,RRAS2 |
| mTOR Signaling | 1.72 | 0.019 | 0 | HRAS,MTOR,RPS6,RRAS2 |
| AMPK Signaling | 1.7 | 0.0188 | #NUM! | CHRNA4,MTOR,PFKP,PPM1A |
| ErbB4 Signaling | 1.38 | 0.0299 | #NUM! | HRAS,RRAS2 |

SM vs. SV comparison

| Ingenuity Canonical Pathways | -log (p-value) | Ratio | z-score | Molecules |
| --- | --- | --- | --- | --- |
| Gluconeogenesis I | 4.2 | 0.154 | -2 | GPI,MDH1,ME1,ME3 |
| Superpathway of D-myo-inositol (1,4,5)-trisphosphate Metabolism | 3.04 | 0.13 | #NUM! | INPP1,ITPKA,SYNJ1 |
| GDP-mannose Biosynthesis | 2.99 | 0.333 | #NUM! | GPI,PMM1 |
| Oxidative Phosphorylation | 2.64 | 0.0459 | -0.447 | COX17,CYB5A,NDUFA9,NDUFS8,UQCRQ |
| Mitochondrial Dysfunction | 2.48 | 0.0351 | #NUM! | COX17,CYB5A,HSD17B10,NDUFA9,NDUFS8,UQCRQ |
| Purine Nucleotides De Novo Biosynthesis II | 2.43 | 0.182 | #NUM! | GART,GMPS |
| Semaphorin Neuronal Repulsive Signaling Pathway | 2.19 | 0.036 | 1.342 | DPYSL3,DPYSL4,GSK3B,GUCY1B1,PPP1CB |
| Superpathway of Citrulline Metabolism | 2.16 | 0.133 | #NUM! | ASL,LOC102724788/PRODH |
| D-myo-inositol (1,4,5)-trisphosphate Degradation | 2.05 | 0.118 | #NUM! | INPP1,SYNJ1 |
| Integrin Signaling | 2.02 | 0.0282 | -1 | ASAP1,CAPN5,GSK3B,NCK2,PPP1CB,TSPAN7 |
| D-myo-inositol (1,3,4)-trisphosphate Biosynthesis | 2.01 | 0.111 | #NUM! | ITPKA,SYNJ1 |
| EIF2 Signaling | 1.92 | 0.0268 | -1 | EIF3M,GSK3B,PPP1CB,RPL10,RPS15,RPS6 |
| Synaptogenesis Signaling Pathway | 1.77 | 0.0224 | 0 | AP2B1,CACNB3,CADM1,CAMK2B,GSK3B,STX1B,YKT6 |
| Alanine Degradation III | 1.77 | 0.5 | #NUM! | GPT |
| Alanine Biosynthesis II | 1.77 | 0.5 | #NUM! | GPT |
| TCA Cycle II (Eukaryotic) | 1.76 | 0.0833 | #NUM! | MDH1,SUCLG1 |
| Glycolysis I | 1.7 | 0.0769 | #NUM! | GPI,PFKM |
| p70S6K Signaling | 1.63 | 0.031 | #NUM! | GNAQ,PPP2R1A,RPS6,YWHAZ |
| Clathrin-mediated Endocytosis Signaling | 1.62 | 0.0259 | #NUM! | AP2B1,CLTB,CSNK2B,SH3GL2,SYNJ1 |
| Proline Degradation | 1.6 | 0.333 | #NUM! | LOC102724788/PRODH |
| HIF1α Signaling | 1.52 | 0.0244 | -2 | CAMK2B,GPI,Ldha/RGD1562690,RAN,RPS6 |
| PRPP Biosynthesis I | 1.48 | 0.25 | #NUM! | PRPS1 |
| Sirtuin Signaling Pathway | 1.43 | 0.0206 | -0.816 | GSK3B,MAP1LC3A,NDUFA9,NDUFS8,PFKM,TUBA8 |
| Creatine-phosphate Biosynthesis | 1.38 | 0.2 | #NUM! | CKMT1A/CKMT1B |
| Citrulline-Nitric Oxide Cycle | 1.38 | 0.2 | #NUM! | ASL |
| tRNA Charging | 1.37 | 0.0513 | #NUM! | MARS1,SARS2 |
| Actin Cytoskeleton Signaling | 1.36 | 0.022 | -1 | BRK1,CYFIP1,EZR,PPP1CB,TRIO |
| Pyruvate Fermentation to Lactate | 1.31 | 0.167 | #NUM! | Ldha/RGD1562690 |
| Dopamine-DARPP32 Feedback in cAMP Signaling | 1.31 | 0.0245 | 2 | GNAQ,GUCY1B1,PPP1CB,PPP2R1A |
| Arginine Biosynthesis IV | 1.31 | 0.167 | #NUM! | ASL |

PM vs. SV comparison

| Ingenuity Canonical Pathways | -log (p-value) | Ratio | z-score | Molecules |
| --- | --- | --- | --- | --- |
| EIF2 Signaling | 10.3 | 0.0625 | -2.714 | EIF3H,EIF5,HRAS,HSPA5,RPL10,RPL17,RPL18,RPL21,RPL27,RPL7,RPL7A,RPS26,RPS6,WARS1 |
| Oxidative Phosphorylation | 3.35 | 0.0459 | -0.447 | ATP5PO,NDUFB11,NDUFB6,NDUFS8,UQCRC1 |
| Mitochondrial Dysfunction | 2.48 | 0.0292 | #NUM! | ATP5PO,NDUFB11,NDUFB6,NDUFS8,UQCRC1 |
| Superpathway of Citrulline Metabolism | 2.47 | 0.133 | #NUM! | ASL,LOC102724788/PRODH |
| G Beta Gamma Signaling | 2.24 | 0.0328 | -1 | CACNG2,GNA13,GNA14,HRAS |
| D-mannose Degradation | 2.23 | 1 | #NUM! | MPI |
| Ephrin B Signaling | 2.06 | 0.0417 | #NUM! | GNA13,GNA14,HRAS |
| Fatty Acid β-oxidation I | 1.8 | 0.0606 | #NUM! | ACSL1,HADH |
| Regulation of eIF4 and p70S6K Signaling | 1.79 | 0.0241 | #NUM! | EIF3H,HRAS,RPS26,RPS6 |
| Proline Degradation | 1.76 | 0.333 | #NUM! | LOC102724788/PRODH |
| 5-aminoimidazole Ribonucleotide Biosynthesis I | 1.76 | 0.333 | #NUM! | GART |
| Nucleotide Excision Repair Pathway | 1.75 | 0.0571 | #NUM! | POLR2B,RAD23B |
| Protein Ubiquitination Pathway | 1.66 | 0.0183 | #NUM! | DNAJC5,HSPA4L,HSPA5,PSMA6,PSMC6 |
| Acetate Conversion to Acetyl-CoA | 1.64 | 0.25 | #NUM! | ACSL1 |
| Synaptic Long Term Depression | 1.6 | 0.0212 | -1 | CACNG2,GNA13,GNA14,HRAS |
| NRF2-mediated Oxidative Stress Response | 1.6 | 0.0212 | #NUM! | AKR7A2,CBR1,DNAJC5,HRAS |
| Creatine-phosphate Biosynthesis | 1.54 | 0.2 | #NUM! | CKMT1A/CKMT1B |
| Serine Biosynthesis | 1.54 | 0.2 | #NUM! | PSAT1 |
| Citrulline-Nitric Oxide Cycle | 1.54 | 0.2 | #NUM! | ASL |
| HIF1α Signaling | 1.49 | 0.0195 | -2 | HRAS,HSPA5,Ldha/RGD1562690,RPS6 |
| Pyruvate Fermentation to Lactate | 1.46 | 0.167 | #NUM! | Ldha/RGD1562690 |
| mTOR Signaling | 1.46 | 0.019 | #NUM! | EIF3H,HRAS,RPS26,RPS6 |
| Arginine Biosynthesis IV | 1.46 | 0.167 | #NUM! | ASL |
| GDP-mannose Biosynthesis | 1.46 | 0.167 | #NUM! | MPI |
| Superpathway of Serine and Glycine Biosynthesis I | 1.4 | 0.143 | #NUM! | PSAT1 |
| Aspartate Degradation II | 1.4 | 0.143 | #NUM! | MDH1 |
| EGF Signaling | 1.39 | 0.0364 | #NUM! | CSNK2B,HRAS |
| Glutamate Receptor Signaling | 1.36 | 0.0351 | #NUM! | SLC17A7,SLC1A3 |

Supplementary Table 3: Ingenuity Pathway Analysis (IPA) of phosphoproteomics

Significant canonical pathways of phosphoproteomics analyzed by IPA, arranged by groups’ comparisons.

PV vs. SV comparison

| Ingenuity Canonical Pathways | -log (p-value) | Ratio | z-score | Molecules |
| --- | --- | --- | --- | --- |
| Synaptogenesis Signaling Pathway | 5.22 | 0.0417 | 1.732 | ADCY9,BRAF,CACNB4,CAMK2A,ITSN1,MAP1B,MAPT,RAF1,STX1B,SYN1,SYN3,SYNGAP1,WASF1 |
| Actin Cytoskeleton Signaling | 5.11 | 0.0485 | 0.632 | APC,CYFIP1,GIT1,MPRIP,MYH7,RAF1,ROCK2,SLC9A1,SSH2,TIAM2,WASF1 |
| Semaphorin Neuronal Repulsive Signaling Pathway | 4.39 | 0.0576 | -2.121 | CRMP1,DPYSL2,DPYSL3,DPYSL4,DPYSL5,MAPT,MPRIP,ROCK2 |
| Protein Kinase A Signaling | 3.51 | 0.03 | 1.508 | ADCY9,ADD2,ANAPC1,BRAF,CAMK2A,GNG10,GYS1,PPP1R7,PTPRD,RAF1,ROCK2,SMAD3 |
| Iron homeostasis signaling pathway | 2.8 | 0.0438 | #NUM! | FTH1,Hba-a1/Hba-a2,JAK2,SLC46A1,SMAD3,STEAP3 |
| Insulin Receptor Signaling | 2.77 | 0.0432 | 0.816 | GYS1,IRS2,JAK2,PPP1R7,RAF1,TSC2 |
| Ephrin Receptor Signaling | 2.76 | 0.037 | 0.816 | GNG10,ITSN1,JAK2,MAP4K4,RAF1,ROCK2,SORBS1 |
| Signaling by Rho Family GTPases | 2.65 | 0.0316 | 1.89 | ARHGEF2,CDC42EP4,CYFIP1,GNG10,RAF1,ROCK2,SLC9A1,WASF1 |
| Gαs Signaling | 2.53 | 0.0467 | 2 | ADCY9,ADD2,BRAF,GNG10,RAPGEF2 |
| Reelin Signaling in Neurons | 2.29 | 0.041 | -0.447 | ARHGEF2,CAMK2A,MAP1B,MAPT,WASF1 |
| RhoA Signaling | 2.27 | 0.0407 | 1.342 | CDC42EP4,MPRIP,RAPGEF2,ROCK2,WASF1 |
| Chemokine Signaling | 2.22 | 0.05 | 0 | CAMK2A,MPRIP,RAF1,ROCK2 |
| α-Adrenergic Signaling | 1.96 | 0.0421 | #NUM! | ADCY9,GNG10,GYS1,RAF1 |
| CDK5 Signaling | 1.77 | 0.037 | 1 | ADCY9,MAPT,PPP1R7,RAF1 |
| Melatonin Signaling | 1.56 | 0.0417 | #NUM! | BRAF,CAMK2A,RAF1 |
| Ephrin B Signaling | 1.56 | 0.0417 | #NUM! | GNG10,ITSN1,ROCK2 |
| Ferroptosis Signaling Pathway | 1.55 | 0.0317 | 2 | BRAF,FTH1,RAF1,STEAP3 |
| RhoGDI Signaling | 1.53 | 0.0265 | #NUM! | ARHGEF2,GNG10,GRIP1,ROCK2,WASF1 |
| PPARα/RXRα Activation | 1.52 | 0.0262 | 0.447 | ADCY9,JAK2,MAP4K4,RAF1,SMAD3 |
| G Protein Signaling Mediated by Tubby | 1.49 | 0.0645 | #NUM! | GNG10,JAK2 |
| Acetate Conversion to Acetyl-CoA | 1.45 | 0.25 | #NUM! | ACSS2 |
| Calcium Signaling | 1.4 | 0.0243 | 1 | CACNB4,CAMK2A,CAMKK1,CAMKK2,MYH7 |
| DNA Methylation and Transcriptional Repression Signaling | 1.4 | 0.0571 | #NUM! | MECP2,SUDS3 |
| HIPPO signaling | 1.37 | 0.0353 | #NUM! | DLG2,PPP1R7,SMAD3 |
| Corticotropin Releasing Hormone Signaling | 1.36 | 0.0276 | 1 | ADCY9,BRAF,CACNB4,RAF1 |
| Creatine-phosphate Biosynthesis | 1.35 | 0.2 | #NUM! | MAP4K4 |
| Myo-inositol Biosynthesis | 1.35 | 0.2 | #NUM! | ISYNA1 |
| AMPK Signaling | 1.34 | 0.0235 | #NUM! | CAMKK2,GYS1,IRS2,PPM1H,TSC2 |

PM vs. PV comparison

| Ingenuity Canonical Pathways | -log (p-value) | Ratio | z-score | Molecules |
| --- | --- | --- | --- | --- |
| Synaptogenesis Signaling Pathway | 8.55 | 0.0513 | 2.5 | AP2A1,BRAF,CAMK2A,CAMK2B,CAMK2G,DLG4,GRM5,MAP1B,MAPK3,MAPT,PRKAR2B,PRKCE,STX1B,SYN1,SYT2,WASF1 |
| Melatonin Signaling | 5.86 | 0.0972 | 0 | BRAF,CAMK2A,CAMK2B,CAMK2G,MAPK3,PRKAR2B,PRKCE |
| Protein Kinase A Signaling | 5.54 | 0.035 | 1.387 | BRAF,CAMK2A,CAMK2B,CAMK2G,GNG10,H1f0,H1f4,MAPK3,PPP1R1B,PPP1R7,PRKAR2B,PRKCE,PTPRN,ROCK2 |
| Reelin Signaling in Neurons | 5.31 | 0.0656 | 2.121 | CAMK2A,CAMK2B,CAMK2G,MAP1B,MAP3K10,MAPK3,MAPT,WASF1 |
| Synaptic Long Term Potentiation | 5.13 | 0.062 | 2.121 | CAMK2A,CAMK2B,CAMK2G,GRM5,MAPK3,PPP1R7,PRKAR2B,PRKCE |
| Semaphorin Neuronal Repulsive Signaling Pathway | 4.9 | 0.0576 | 0 | CRMP1,DPYSL2,DPYSL4,DPYSL5,MAPT,PIP5K1C,PRKAR2B,ROCK2 |
| Calcium Signaling | 4.48 | 0.0437 | 0.707 | ATP2A2,ATP2B3,ATP2B4,CAMK2A,CAMK2B,CAMK2G,HDAC1,MAPK3,PRKAR2B |
| Pyridoxal 5'-phosphate Salvage Pathway | 3.8 | 0.0758 | 2.236 | BRAF,MAPK3,PRKAA1,PRKCE,PRPF4B |
| G-Protein Coupled Receptor Signaling | 3.54 | 0.0328 | #NUM! | BRAF,CAMK2A,CAMK2B,CAMK2G,GRM5,MAPK3,PRKAR2B,PRKCE,RGS12 |
| Sirtuin Signaling Pathway | 3.35 | 0.0309 | -0.333 | ATP5F1B,H1f0,H1f4,MAPK3,PCK2,PDHA1,PRKAA1,TP53BP1,TRIM28 |
| Insulin Secretion Signaling Pathway | 3.2 | 0.0328 | 2.121 | CAMK2A,CAMK2B,CAMK2G,EIF4G3,MAPK3,PDHA1,PRKAR2B,PRKCE |
| Salvage Pathways of Pyrimidine Ribonucleotides | 3.01 | 0.051 | 2.236 | BRAF,MAPK3,PRKAA1,PRKCE,PRPF4B |
| HIF1α Signaling | 2.96 | 0.0341 | 1.89 | BRAF,CAMK2A,CAMK2B,CAMK2G,MAPK3,PRKCE,STUB1 |
| Gαs Signaling | 2.84 | 0.0467 | 2 | BRAF,GNG10,MAPK3,PRKAR2B,RAPGEF2 |
| CDK5 Signaling | 2.82 | 0.0463 | 1.342 | MAPK3,MAPT,PPP1R1B,PPP1R7,PRKAR2B |
| cAMP-mediated signaling | 2.69 | 0.0306 | 1.134 | BRAF,CAMK2A,CAMK2B,CAMK2G,MAPK3,PRKAR2B,RGS12 |
| Ephrin B Signaling | 2.63 | 0.0556 | #NUM! | CAP1,GNG10,MAPK3,ROCK2 |
| CREB Signaling in Neurons | 2.63 | 0.0201 | 1.508 | BMPR2,CAMK2A,CAMK2B,CAMK2G,CCKBR,GNG10,GPR158,GRM5,MAPK3,PRKAR2B,PRKCE,SSTR2 |
| PPARα/RXRα Activation | 2.43 | 0.0314 | 0.816 | BMPR2,MAP4K4,MAPK3,NCOR2,PRKAA1,PRKAR2B |
| Axonal Guidance Signaling | 2.29 | 0.0202 | #NUM! | DPYSL2,DPYSL5,GIT1,GNG10,KLC1,MAPK3,PRKAR2B,PRKCE,ROCK2,RTN4 |
| nNOS Signaling in Neurons | 2.24 | 0.0638 | #NUM! | CAMK2A,DLG4,PRKCE |
| α-Adrenergic Signaling | 2.2 | 0.0421 | #NUM! | GNG10,MAPK3,PRKAR2B,PRKCE |
| Gαq Signaling | 2.12 | 0.0318 | 0 | GNG10,GRM5,MAPK3,PRKCE,ROCK2 |
| Actin Cytoskeleton Signaling | 2.07 | 0.0264 | 0.816 | ARHGAP35,GIT1,MAPK3,PIP5K1C,ROCK2,WASF1 |
| Dopamine-DARPP32 Feedback in cAMP Signaling | 2.06 | 0.0307 | 1 | ATP2A2,PPP1R1B,PPP1R7,PRKAR2B,PRKCE |
| 3-phosphoinositide Biosynthesis | 2.03 | 0.0301 | 0.447 | ATP1A1,PIP5K1C,PPP1R1B,PPP1R7,PTPRN |
| Glutamate Receptor Signaling | 2.01 | 0.0526 | #NUM! | DLG4,GRM5,SLC1A2 |
| NGF Signaling | 1.93 | 0.0351 | #NUM! | MAP3K10,MAPK3,ROCK2,SMPD3 |
| Signaling by Rho Family GTPases | 1.86 | 0.0237 | 1.342 | GNG10,MAP3K10,MAPK3,PIP5K1C,ROCK2,WASF1 |
| G Beta Gamma Signaling | 1.83 | 0.0328 | 2 | GNG10,MAPK3,PRKAR2B,PRKCE |
| Gαi Signaling | 1.79 | 0.032 | #NUM! | GNG10,MAPK3,PRKAR2B,RGS12 |
| ERK/MAPK Signaling | 1.69 | 0.0248 | 2.236 | BRAF,MAPK3,PPP1R7,PRKAR2B,PRKCE |
| Dopamine Receptor Signaling | 1.66 | 0.039 | #NUM! | PPP1R1B,PPP1R7,PRKAR2B |
| BMP signaling pathway | 1.55 | 0.0353 | #NUM! | BMPR2,MAPK3,PRKAR2B |
| DNA Methylation and Transcriptional Repression Signaling | 1.53 | 0.0571 | #NUM! | HDAC1,SUDS3 |
| GABA Receptor Signaling | 1.43 | 0.0316 | #NUM! | AP2A1,GPHN,SLC6A11 |
| Apoptosis Signaling | 1.37 | 0.03 | #NUM! | MAP4K4,MAPK3,PRKCE |
| Neuregulin Signaling | 1.32 | 0.0286 | #NUM! | DLG4,MAPK3,PRKCE |
| PFKFB4 Signaling Pathway | 1.31 | 0.0435 | #NUM! | MAPK3,PRKAR2B |

SM vs. SV comparison

| Ingenuity Canonical Pathways | -log  (p-value) | Ratio | z-score | Molecules |
| --- | --- | --- | --- | --- |
| Dopamine-DARPP32 Feedback in cAMP Signaling | 7.05 | 0.0613 | -0.707 | ATP2A2,CACNA1A,CREBBP,GRIN2A,KCNJ11,PLCB1,PPP1R1B,PPP1R7,PRKAR1A,PRKCG |
| Synaptogenesis Signaling Pathway | 7.02 | 0.0417 | -0.277 | CAMK2A,CREBBP,DLG4,GRIN2A,ITSN1,MAP1B,MAPT,PRKAR1A,RAF1,SHC3,SRC,STXBP5,SYN1 |
| Synaptic Long Term Potentiation | 5.78 | 0.062 | -0.707 | CAMK2A,CREBBP,GRIN2A,PLCB1,PPP1R7,PRKAR1A,PRKCG,RAF1 |
| Insulin Secretion Signaling Pathway | 5.45 | 0.041 | -0.632 | CACNA1A,CAMK2A,CREBBP,EIF4G3,KCNJ11,PLCB1,PRKAR1A,PRKCG,SRC,SRP14 |
| nNOS Signaling in Neurons | 4.94 | 0.106 | #NUM! | CAMK2A,DLG2,DLG4,GRIN2A,PRKCG |
| Reelin Signaling in Neurons | 4.9 | 0.0574 | -1.134 | ARHGEF12,ARHGEF2,CAMK2A,GRIN2A,MAP1B,MAPT,SRC |
| Protein Kinase A Signaling | 4.31 | 0.0275 | -1 | CAMK2A,CREBBP,GNG3,MYLK,PLCB1,PPP1R1B,PPP1R7,PRKAR1A,PRKCG,PTPRZ1,RAF1 |
| Calcium Signaling | 4.29 | 0.0388 | 0.378 | ATP2A2,ATP2B4,CACNA1A,CAMK2A,CREBBP,GRIN2A,MYH7,PRKAR1A |
| CDK5 Signaling | 4.19 | 0.0556 | 1.633 | CACNA1A,MAPT,PPP1R1B,PPP1R7,PRKAR1A,RAF1 |
| Axonal Guidance Signaling | 4.14 | 0.0243 | #NUM! | ARHGEF12,DPYSL2,DPYSL5,GIT1,GNG3,ITSN1,PLCB1,PRKAR1A,PRKCG,RAF1,RTN4,SRGAP3 |
| Melatonin Signaling | 4.03 | 0.0694 | 0 | CAMK2A,PLCB1,PRKAR1A,PRKCG,RAF1 |
| G Beta Gamma Signaling | 3.9 | 0.0492 | 0 | CACNA1A,GNG3,PRKAR1A,PRKCG,RAF1,SRC |
| P2Y Purigenic Receptor Signaling Pathway | 3.8 | 0.0472 | -0.447 | CREBBP,GNG3,PLCB1,PRKAR1A,PRKCG,RAF1 |
| p70S6K Signaling | 3.77 | 0.0465 | -0.816 | MAPT,PLCB1,PRKCG,RAF1,RPS6,SRC |
| Semaphorin Neuronal Repulsive Signaling Pathway | 3.59 | 0.0432 | -2.449 | ARHGEF12,CSPG5,DPYSL2,DPYSL5,MAPT,PRKAR1A |
| Phospholipase C Signaling | 3.53 | 0.0301 | -1.89 | ARHGEF12,ARHGEF2,CREBBP,GNG3,PLCB1,PRKCG,RAF1,SRC |
| G-Protein Coupled Receptor Signaling | 3.44 | 0.0292 | #NUM! | CAMK2A,CREBBP,PLCB1,PRKAR1A,PRKCG,RAF1,RGS12,SRC |
| Neuregulin Signaling | 3.27 | 0.0476 | -1.342 | DLG4,PRKCG,RAF1,RPS6,SRC |
| Gαi Signaling | 2.92 | 0.04 | -1 | GNG3,PRKAR1A,RAF1,RGS12,SRC |
| Ephrin Receptor Signaling | 2.89 | 0.0317 | 0 | CREBBP,GNG3,GRIN2A,ITSN1,RAF1,SRC |
| Calcium Transport I | 2.76 | 0.2 | #NUM! | ATP2A2,ATP2B4 |
| ERK/MAPK Signaling | 2.74 | 0.0297 | -0.816 | CREBBP,PPP1R7,PRKAR1A,PRKCG,RAF1,SRC |
| α-Adrenergic Signaling | 2.51 | 0.0421 | #NUM! | GNG3,PRKAR1A,PRKCG,RAF1 |
| D-myo-inositol-5-phosphate Metabolism | 2.49 | 0.0318 | 0 | ATP1A1,ATP1A2,PLCB1,PPP1R1B,PPP1R7 |
| cAMP-mediated signaling | 2.47 | 0.0262 | -1.633 | CAMK2A,CREBBP,PRKAR1A,RAF1,RGS12,SRC |
| NRF2-mediated Oxidative Stress Response | 2.15 | 0.0265 | 0 | CREBBP,DNAJC6,FTH1,PRKCG,RAF1 |
| Wnt/Ca+ pathway | 2.15 | 0.0484 | #NUM! | CAMK2A,CREBBP,PLCB1 |
| Ferroptosis Signaling Pathway | 2.08 | 0.0317 | 1 | FTH1,H2BC5,RAF1,STEAP3 |
| Integrin Signaling | 1.94 | 0.0235 | 1 | ARHGAP5,GIT1,MYLK,RAF1,SRC |
| CREB Signaling in Neurons | 1.87 | 0.0151 | 0 | CACNA1A,CAMK2A,CREBBP,GNG3,GRIN2A,PLCB1,PRKAR1A,PRKCG,RAF1 |
| Actin Cytoskeleton Signaling | 1.83 | 0.022 | 0 | ARHGEF12,GIT1,MYH7,MYLK,RAF1 |
| G Protein Signaling Mediated by Tubby | 1.79 | 0.0645 | #NUM! | GNG3,PLCB1 |
| BMP signaling pathway | 1.78 | 0.0353 | #NUM! | CREBBP,PRKAR1A,RAF1 |
| Gαq Signaling | 1.75 | 0.0255 | #NUM! | GNG3,PLCB1,PRKCG,RAF1 |
| Synaptic Long Term Depression | 1.49 | 0.0212 | 0 | CACNA1A,PLCB1,PRKCG,RAF1 |
| PPARα/RXRα Activation | 1.48 | 0.0209 | #NUM! | CREBBP,PLCB1,PRKAR1A,RAF1 |
| RAR Activation | 1.46 | 0.0206 | #NUM! | CREBBP,PRKAR1A,PRKCG,SRC |

PM vs. SV comparison

| Ingenuity Canonical Pathways | -log (p-value) | Ratio | z-score | Molecules |
| --- | --- | --- | --- | --- |
| Synaptogenesis Signaling Pathway | 12.7 | 0.0929 | 2.117 | AKT1,BAD,CACNA1B,CACNB4,CAMK2A,CAMK2B,CDH10,CDH8,CTNND1,DLG4,EFNB1,GRIN2A,GRIN2B,GRM5,ITSN1,MAP1B,MAPK1,MAPK3,MAPT,PRKAR1A,RAF1,RASGRF1,SHC3,SNAP25,STX1A,STX1B,STXBP5,SYNGAP1,SYT2 |
| Calcium Signaling | 6.92 | 0.0825 | 2.324 | ATP2A2,ATP2B2,ATP2B4,CACNA1B,CACNA1E,CACNB4,CAMK2A,CAMK2B,CAMKK1,CHRNA4,GRIN2A,GRIN2B,HDAC2,MAPK1,MAPK3,MYH7,PRKAR1A |
| Reelin Signaling in Neurons | 6.7 | 0.107 | 1.387 | AKT1,ARHGEF11,ARHGEF12,ARHGEF2,CAMK2A,CAMK2B,GRIN2A,GRIN2B,MAP1B,MAPK1,MAPK3,MAPK8IP3,MAPT |
| Synaptic Long Term Potentiation | 6.41 | 0.101 | 0.832 | CAMK2A,CAMK2B,GRIN2A,GRIN2B,GRM5,MAPK1,MAPK3,PLCB1,PLCL1,PPP1R7,PRKAR1A,PRKCG,RAF1 |
| Melatonin Signaling | 5.39 | 0.125 | 0 | CAMK2A,CAMK2B,MAPK1,MAPK3,PLCB1,PLCL1,PRKAR1A,PRKCG,RAF1 |
| Actin Cytoskeleton Signaling | 4.36 | 0.0617 | 0.832 | APC,ARHGAP35,ARHGEF12,CYFIP1,GIT1,MAPK1,MAPK3,MYH7,RAF1,ROCK2,SLC9A1,SSH2,TIAM2,TRIO |
| Glutamate Receptor Signaling | 4.26 | 0.123 | #NUM! | DLG4,GRIN2A,GRIN2B,GRIP1,GRM5,SLC1A2,SLC1A4 |
| Protein Kinase A Signaling | 4.12 | 0.0475 | 1.147 | ADD1,ADD3,ANAPC1,BAD,CAMK2A,CAMK2B,H1f0,MAPK1,MAPK3,PLCB1,PLCL1,PPP1R1B,PPP1R7,PRKAR1A,PRKCG,PTPRD,PTPRN,RAF1,ROCK2 |
| Dopamine-DARPP32 Feedback in cAMP Signaling | 3.89 | 0.0675 | 0 | ATP2A2,CACNA1E,CAMKK1,GRIN2A,GRIN2B,PLCB1,PLCL1,PPP1R1B,PPP1R7,PRKAR1A,PRKCG |
| nNOS Signaling in Neurons | 3.82 | 0.128 | 0 | CAMK2A,DLG2,DLG4,GRIN2A,GRIN2B,PRKCG |
| Insulin Receptor Signaling | 3.81 | 0.0719 | 1.265 | ACLY,AKT1,BAD,JAK2,MAPK1,MAPK3,PPP1R7,PRKAR1A,RAF1,TSC2 |
| G Beta Gamma Signaling | 3.56 | 0.0738 | 2.333 | AKT1,CACNA1B,CACNA1E,CACNB4,MAPK1,MAPK3,PRKAR1A,PRKCG,RAF1 |
| G-Protein Coupled Receptor Signaling | 3.5 | 0.0511 | #NUM! | AKT1,CAMK2A,CAMK2B,GABBR2,GRM5,MAPK1,MAPK3,PLCB1,PRKAR1A,PRKCG,RAF1,RAP1GAP,RGS12,SYNGAP1 |
| p70S6K Signaling | 3.38 | 0.0698 | 1.667 | AKT1,BAD,MAPK1,MAPK3,MAPT,PLCB1,PLCL1,PRKCG,RAF1 |
| Synaptic Long Term Depression | 3.33 | 0.0582 | 1.265 | CACNA1B,CACNA1E,CACNB4,GRM5,MAPK1,MAPK3,PLCB1,PLCL1,PRKCG,RAF1,SMARCC2 |
| NRF2-mediated Oxidative Stress Response | 3.33 | 0.0582 | 1.134 | AKT1,DNAJA4,DNAJC6,FTH1,MAPK1,MAPK3,PRKCG,RAF1,SOD1,STIP1,USP14 |
| Ephrin Receptor Signaling | 3.33 | 0.0582 | 1.897 | AKT1,EFNB1,GRIN2A,GRIN2B,ITSN1,JAK2,MAPK1,MAPK3,RAF1,ROCK2,SORBS1 |
| Calcium Transport I | 3.25 | 0.3 | #NUM! | ATP2A2,ATP2B2,ATP2B4 |
| Semaphorin Neuronal Repulsive Signaling Pathway | 3.15 | 0.0647 | -1.667 | AKT1,ARHGEF12,BCAN,CRMP1,CSPG5,DPYSL2,MAPT,PRKAR1A,ROCK2 |
| Insulin Secretion Signaling Pathway | 2.93 | 0.0492 | 1.732 | CACNA1E,CAMK2A,CAMK2B,EIF4G3,JAK2,MAPK1,MAPK3,PLCB1,PLCL1,PRKAR1A,PRKCG,SNAP25 |
| Ferroptosis Signaling Pathway | 2.8 | 0.0635 | 0.707 | ACACA,FTH1,G3BP1,MAPK1,MAPK3,RAF1,USP7,VDAC2 |
| P2Y Purigenic Receptor Signaling Pathway | 2.78 | 0.063 | 1.414 | AKT1,MAPK1,MAPK3,PLCB1,PLCL1,PRKAR1A,PRKCG,RAF1 |
| IGF-1 Signaling | 2.66 | 0.0673 | 1.89 | AKT1,BAD,JAK2,MAPK1,MAPK3,PRKAR1A,RAF1 |
| Neuregulin Signaling | 2.63 | 0.0667 | 1.134 | AKT1,BAD,DLG4,MAPK1,MAPK3,PRKCG,RAF1 |
| Axonal Guidance Signaling | 2.6 | 0.0364 | #NUM! | AKT1,ARHGEF11,ARHGEF12,DPYSL2,EFNB1,GIT1,ITSN1,KLC1,MAPK1,MAPK3,PLCB1,PLCL1,PRKAR1A,PRKCG,RAF1,ROCK2,RTN4,SRGAP3 |
| CDK5 Signaling | 2.57 | 0.0648 | 0.378 | MAPK1,MAPK3,MAPT,PPP1R1B,PPP1R7,PRKAR1A,RAF1 |
| Sirtuin Signaling Pathway | 2.31 | 0.0412 | 0.632 | ACLY,AKT1,ATG9A,H1f0,MAPK1,MAPK3,PCK2,SLC25A4,SOD1,TP53BP1,TRIM28,VDAC2 |
| ErbB2-ErbB3 Signaling | 2.28 | 0.0769 | 2.236 | AKT1,BAD,MAPK1,MAPK3,RAF1 |
| Pyridoxal 5'-phosphate Salvage Pathway | 2.25 | 0.0758 | 2.236 | MAPK1,MAPK3,PKN1,PNPO,PRPF4B |
| ErbB4 Signaling | 2.22 | 0.0746 | 1.342 | AKT1,MAPK1,MAPK3,PRKCG,RAF1 |
| Gαq Signaling | 2.22 | 0.051 | 0.707 | AKT1,GRM5,MAPK1,MAPK3,PLCB1,PRKCG,RAF1,ROCK2 |
| Gαi Signaling | 2.22 | 0.056 | 0.378 | GABBR2,MAPK1,MAPK3,PRKAR1A,RAF1,RAP1GAP,RGS12 |
| GABA Receptor Signaling | 2.21 | 0.0632 | #NUM! | CACNA1B,CACNA1E,CACNB4,DNM1,GABBR2,GPHN |
| PPARα/RXRα Activation | 2.21 | 0.0471 | 0 | BMPR2,JAK2,MAPK1,MAPK3,PLCB1,PLCL1,PRKAR1A,RAF1,TGS1 |
| VEGF Signaling | 2.13 | 0.0606 | 2.449 | AKT1,BAD,MAPK1,MAPK3,RAF1,ROCK2 |
| Gα12/13 Signaling | 2.11 | 0.0534 | 1.134 | AKT1,CDH10,CDH8,MAPK1,MAPK3,RAF1,ROCK2 |
| Iron homeostasis signaling pathway | 2.01 | 0.0511 | #NUM! | BMPR2,FTH1,Hba-a1/Hba-a2,JAK2,MAPK1,MAPK3,SKP1 |
| Gαs Signaling | 1.97 | 0.0561 | 0.816 | ADD1,ADD3,MAPK1,MAPK3,PRKAR1A,RAPGEF2 |
| NGF Signaling | 1.84 | 0.0526 | 2.236 | AKT1,MAPK1,MAPK3,RAF1,ROCK2,TRIO |
| BMP signaling pathway | 1.8 | 0.0588 | 1.342 | BMPR2,MAPK1,MAPK3,PRKAR1A,RAF1 |
| CREB Signaling in Neurons | 1.79 | 0.0302 | 1.886 | AKT1,BMPR2,CACNA1B,CACNA1E,CACNB4,CAMK2A,CAMK2B,GABBR2,GRIN2A,GRIN2B,GRM5,MAPK1,MAPK3,PLCB1,PLCL1,PRKAR1A,PRKCG,RAF1 |
| PDGF Signaling | 1.78 | 0.0581 | 1.342 | JAK2,MAPK1,MAPK3,RAF1,SPHK2 |
| Cleavage and Polyadenylation of Pre-mRNA | 1.76 | 0.167 | #NUM! | CSTF2,CSTF3 |
| cAMP-mediated signaling | 1.73 | 0.0393 | 1 | CAMK2A,CAMK2B,GABBR2,MAPK1,MAPK3,PRKAR1A,RAF1,RAP1GAP,RGS12 |
| Superpathway of Inositol Phosphate Compounds | 1.64 | 0.0402 | 1.134 | ATP1A1,ATP1A2,NUDT5,PLCB1,PPP1R1B,PPP1R7,PTPRN,SEC16A |
| α-Adrenergic Signaling | 1.61 | 0.0526 | 1 | MAPK1,MAPK3,PRKAR1A,PRKCG,RAF1 |
| Netrin Signaling | 1.59 | 0.0615 | 2 | CACNA1B,CACNA1E,CACNB4,PRKAR1A |
| Apoptosis Signaling | 1.53 | 0.05 | -2.236 | BAD,LMNA,MAPK1,MAPK3,RAF1 |
| AMPK Signaling | 1.49 | 0.0376 | 0.816 | ACACA,AKT1,CHRNA4,MAPK1,PCK2,PRKAR1A,SMARCC2,TSC2 |
| PI3K/AKT Signaling | 1.39 | 0.038 | 1.134 | AKT1,BAD,JAK2,MAPK1,MAPK3,RAF1,TSC2 |
| Phospholipase C Signaling | 1.37 | 0.0338 | 0.333 | ARHGEF11,ARHGEF12,ARHGEF2,HDAC2,MAPK1,MAPK3,PLCB1,PRKCG,RAF1 |
| Neurotrophin/TRK Signaling | 1.37 | 0.0526 | 2 | AKT1,MAPK1,MAPK3,RAF1 |
| PFKFB4 Signaling Pathway | 1.35 | 0.0652 | #NUM! | MAPK1,MAPK3,PRKAR1A |
| Protein Ubiquitination Pathway | 1.32 | 0.033 | #NUM! | ANAPC1,DNAJC6,PSMD1,PSMD3,SKP1,UBA1,USP14,USP5,USP7 |

#NUM! – no predicted z-score.
